# Supplementary material for: Modeling of full-length Piezo1 suggests importance of the proximal N-terminus for dome structure
Source: Biophys J. 2021 Feb 12;120(8):1343–56. doi: 10.1016/j.bpj.2021.02.003 (PMC8105715; doi:10.1016/j.bpj.2021.02.003)
Supplement: Document S1. Figs. S1–S11 and Tables S1–S6 [file mmc1.pdf]

Biophysical Journal, Volume *120*

Supplemental information

Modeling of full-length Piezo1 suggests importance of the proximal N-terminus for dome structure

Jiehan Chong, Dario De Vecchis, Adam J. Hyman, Oleksandr V. Povstyan, Melanie J. Ludlow, Jian Shi, David J. Beech, and Antreas C. Kalli

Supplementary Table 1: Membrane lipid compositions used for the CG-MD simulations

**Name**	**Extracellular leaflet**				**Cytoplasmic leaflet**				
	POPC	POPE	SM	CHOL	POPC	POPE	POPS	PIP2	CHOL
$CHOL0_{trunc}$	75	20	5	0	70	20	5	5	0
$CHOL5_{trunc}$	70	20	5	5	65	20	5	5	5
$CHOL10_{trunc}$	65	20	5	10	60	20	5	5	10
$CHOL20_{trunc}$	55	20	5	20	50	20	5	5	20
$CHOL30_{trunc}$	45	20	5	30	40	20	5	5	30
$CHOL40_{trunc}$	35	20	5	40	30	20	5	5	40
$CHOL0_{full}$	75	20	5	0	70	20	5	5	0
$CHOL10_{full}$	65	20	5	10	60	20	5	5	10
$CHOL20_{full}$	55	20	5	20	50	20	5	5	20
$CHOL30_{full}$	45	20	5	30	40	20	5	5	30
$CHOL40_{full}$	35	20	5	40	30	20	5	5	40

Lipid composition is expressed in molar percentages

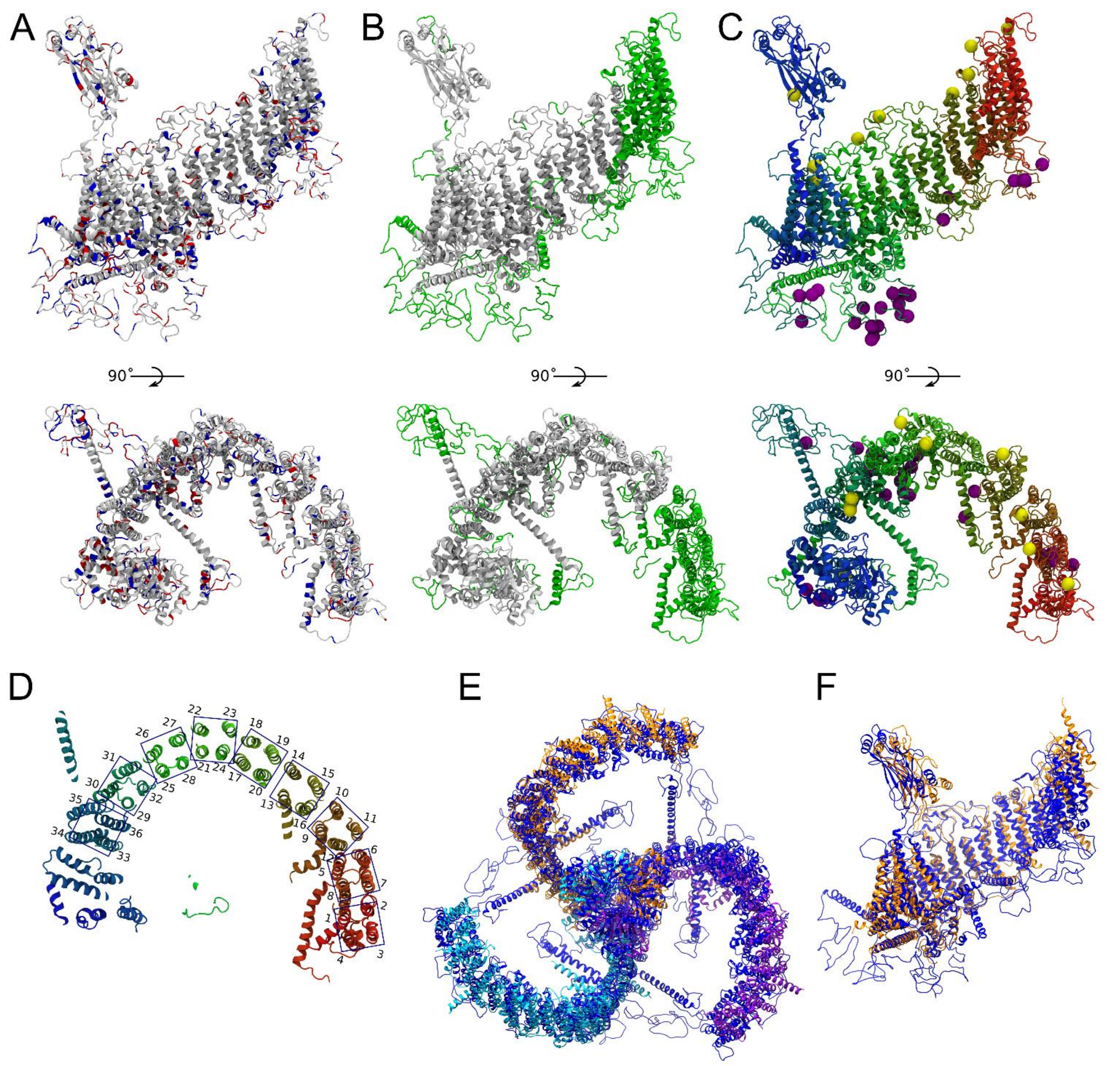

Supplementary Figure 1: Characterization of a single full-length chain Piezo1 mouse model. (A) A single full-length chain is shown as ribbon with positively (His, Lys, Arg) and negatively (Asp, Glu) charged residues colored in blue and red, respectively. **(B)** A single full-length chain is shown as ribbon with modelled missing residues from the PDB 6B3R, highlighted in green. **(C)** A single full-length chain is shown as ribbon and colored rainbow from N-terminal (red) to C-terminal (blue). Spheres are Cα atoms from residues previously identified as exposed to the extracellular side (Myc tagged, yellow) and phosphorylated (purple). Myc tagged residues are: 102, 304, 508, 669, 897, 1071, 1765, 2071, 2075 and 2336. Phosphorylated residues are: 351, 396, 397, 399, 738, 758, 1385, 1389, 1390, 1593, 1600, 1604, 1608, 1610, 1612, 1617, 1626, 1627, 1631, 1640, 1644, 1646 and 1837. **(D)** Cropped visualization of a single Piezo1 full-length chain. The numbering and position of each of the 36 bundles is indicated. **(E)** Structural superposition between the Piezo1 full-length model (blue) and the Piezo2 cryo-EM structure (chains are in orange, purple and cyan; PDB: 6KG7) performed with UCSF Chimera over the Cα atoms. **(F)** A single chain from the Piezo2 cryo-EM structure (orange) and a Piezo1 full-length model superposed with UCSF Chimera over their Cα atoms. The long loops in the cytoplasm (residues 718-781, 1366-1492, 1579-1654, 1808-1951, 542-568, 337-418, 145-182) were not considered in this alignment.

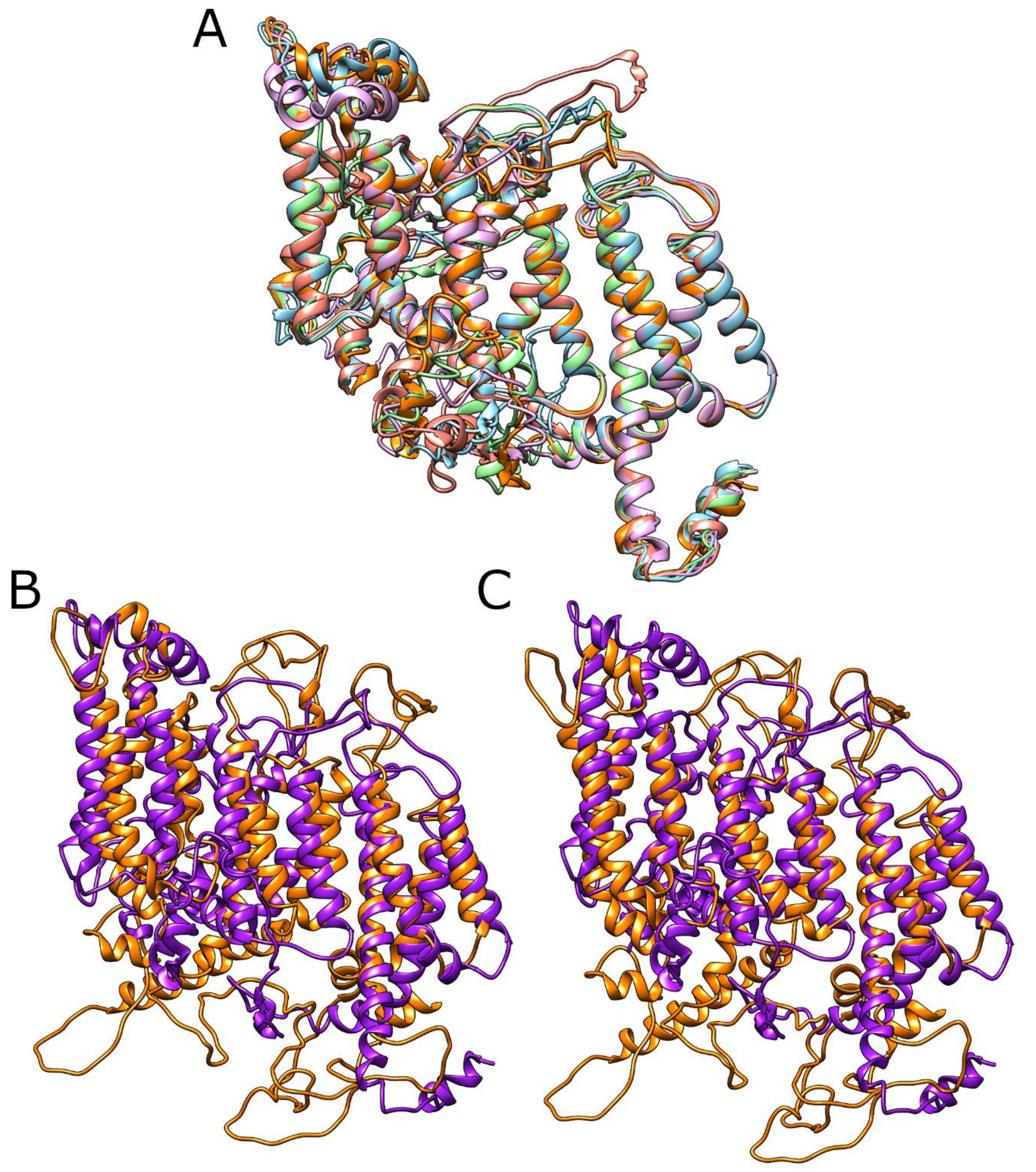

Supplementary Figure 2: Modelling of Piezo1 N-terminal residues by I-TASSER. (A) Top 5 models of Piezo1 N-terminal produced by I-TASSER, superimposed on each other. Model 1 is orange, model 2 is blue, model 3 is pink, model 4 is green, and model 5 is red. **(B)** Top-scoring I-TASSER model (orange) aligned with the corresponding region of our Piezo1$_{full}$ model (purple). All modelled residues (1-576) were used for alignment (RMSD 16.93 Å). **(C)** Top-scoring I-TASSER model (orange) superimposed on our Piezo1$_{full}$ model (purple), with alignment by transmembrane helices only (RMSD of aligned regions 10.18 Å).

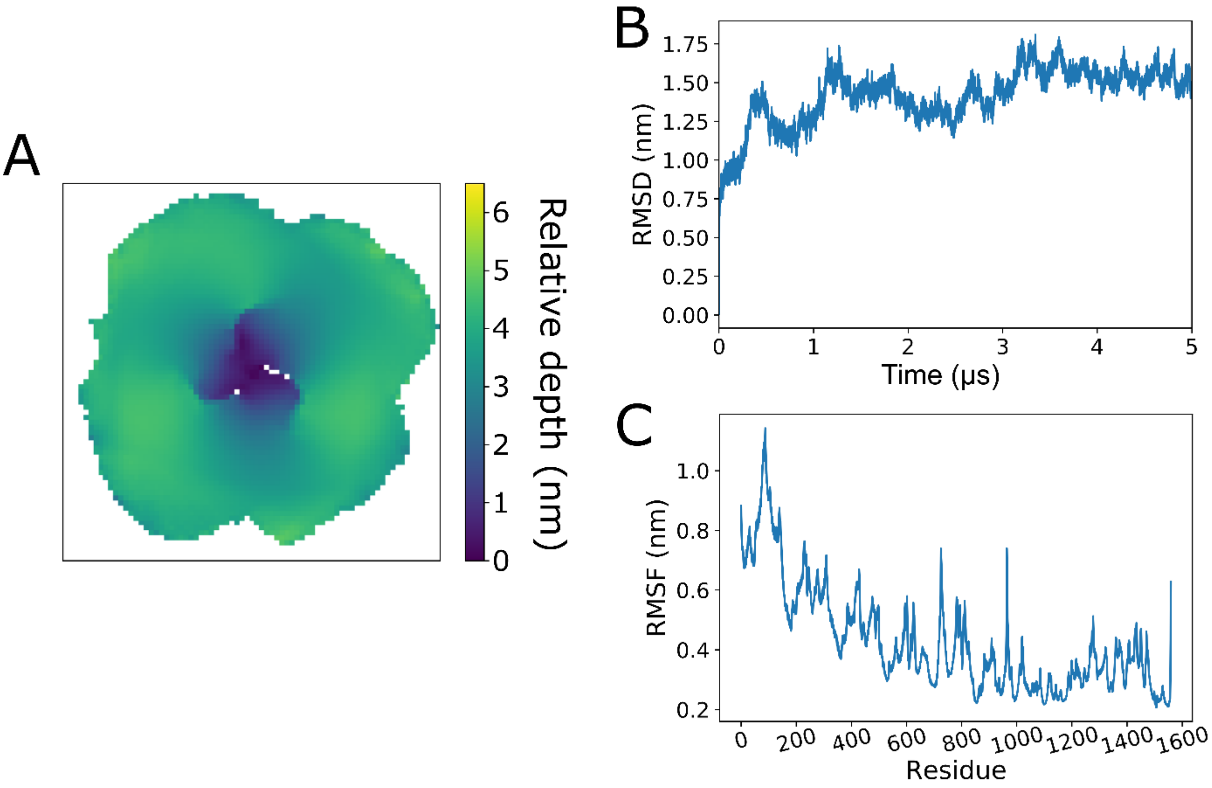

Supplementary Figure 3: (A) Height maps of CG phosphate beads corresponding to the extracellular leaflet in 5 simulations of a Piezo1$_{trunc}$ in a pure PC bilayer, averaged across all repeats. **(B)** RMSD of Piezo1$_{trunc}$ CG beads in a PC bilayer in simulation 1. **(C)** RMSF of Piezo1$_{trunc}$ CG beads in a PC bilayer in simulation 1.

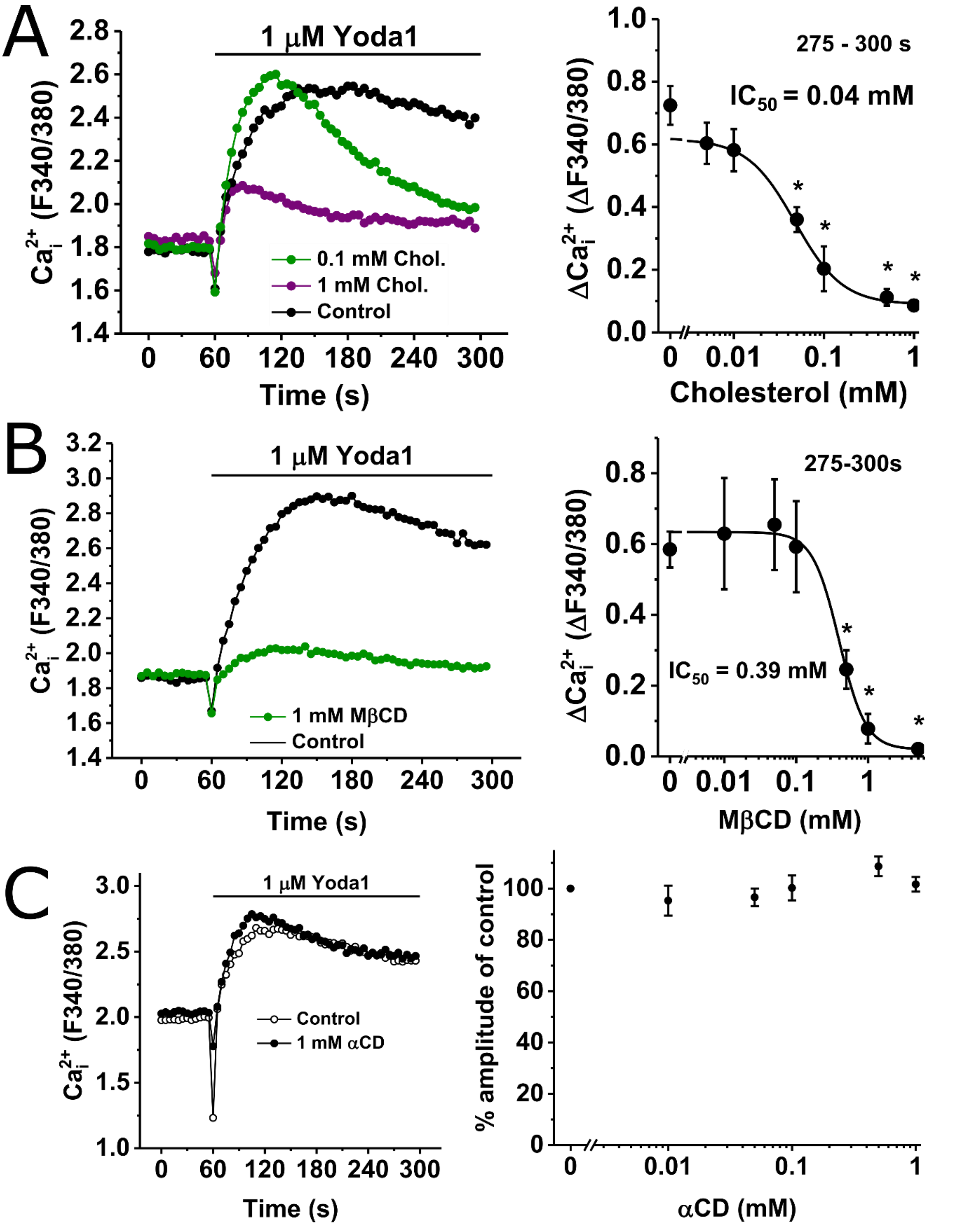

Supplementary Figure 4: Cholesterol concentration modulates Piezo1 activity in HUVECs (A) Left, an example 96-well plate fura-2 measurement of the change in intracellular Ca^{2+} concentration evoked by 1 μM Yoda1 in HUVECs pre-treated with 0 (control), 0.1, and 1 mM cholesterol. Right, mean data for the average amplitude (at 275-300 seconds) of HUVEC responses to Yoda1 with varying doses of cholesterol (0.005-1 mM) displayed as a Hill Equation indicating the 50 % inhibitory effect (IC50) at 0.04 mM. n/N=4/4. (**B**) Left, an example 96-well plate fura-2 measurement of the change in intracellular Ca^{2+} concentration evoked by 1 μM Yoda1 in HUVECs pre-treated with 0 (control) and 1 mM MβCD. Right, mean

data for the average amplitude (at 275-300 seconds) of HUVEC responses to Yoda1 with varying doses of MβCD (5-0.01 mM) displayed as a Hill Equation indicating the 50 % inhibitory effect (IC50) at 0.39 mM. n/N=4/4. **(C)** Left, an example measurement of the change in intracellular Ca^{2+} concentration evoked by 1 μM Yoda1 in HUVECs pre-treated with 0 (control) and 1 mM αCD. Right, mean data of peak amplitudes of HUVEC responses to increasing doses of αCD (1-0.01 mM). n/N=3/4.

Supplementary Table 2: Piezo1$_{trunc}$ residues with cholesterol contacts exceeding cutoff of 60% of maximum contacts.

Y582^{e}	Q703^{e}	D1211^{cef}	M1731^{cef}
V583^{def}	H705^{f}	L1215^{ef}	T1732^{ce}
V589^{def}	Y706^{ef}	M1494^{f}	V1735^{c}
T619^{e}	Y782^{f}	M1495^{f}	E1738^{f}
**Q622**^{bcdef}	L786^{def}	A1499^{f}	M1740^{bcde}
T626^{ef}	S789^{def}	Q1503^{ef}	T1743^{bdf}
L627^{bcde}	F800^{ef}	**F1504**^{bcdef}	K1744^{bcd}
W636^{ef}	H808^{cf}	L1508^{ef}	L1774^{d}
W637^{def}	K811^{cdef}	G1509^{ce}	G1775^{b}
L647^{e}	F986^{e}	T1512^{e}	K1784^{ef}
V650^{e}	**A1019**^{bcdef}	R1671^{cdef}	Y1785^{def}
Y651^{ef}	**T1022**^{bcdef}	T1672^{cef}	F2028^{e}
F655^{ef}	**R1023**^{bcdef}	Y1680^{cef}	W2060^{ef}
F687^{f}	Y1062^{ef}	A1706^{d}	V2141^{f}
A699^{e}	Y1190^{f}	W1717^{f}	W2142^{ef}
L702^{def}	V1208^{ef}	R1728^{cef}	Y2189^{cdef}

The simulated cholesterol concentration in which the residue exceeds cutoff is represented by superscript letters after each residue. Key: b – 5%, c – 10%, d – 20%, e – 30%, f – 40%. Residues which were above cut-off at all simulated cholesterol concentrations are in bold. Residues coinciding with CRAC or CARC motifs are shown in blue. Residues also exceeding cut-off in Piezo1$_{full}$ simulations are underlined.

Supplementary Table 3: Piezo1$_{full}$ residues with cholesterol contacts exceeding cut-off of 60% of maximum contacts.

**L20**^{cdef}	F90^{f}	G265^{ef}	R796^{f}	G1509^{ef}	Q1944^{ef}
**R26**^{cdef}	I105^{ef}	**T313**^{cdef}	Q798^{f}	T1512^{ef}	F1946^{f}
L32^{def}	G106^{f}	W317^{f}	F800^{ef}	V1513^{ef}	V1948^{f}
V33^{ef}	T119^{f}	Y320^{f}	R802^{f}	**R1671**^{cdef}	Y1955^{f}
P42^{cef}	**A123**^{cdef}	D428^{f}	F838^{f}	T1672^{f}	I1988^{e}
W43^{cef}	**P124**^{cdef}	W533^{ef}	F845^{f}	Y1680^{f}	F2028^{e}
**P47**^{cdef}	R141^{f}	Y588^{f}	F936^{f}	A1706^{def}	W2060^{ef}
R58^{def}	R187^{ef}	Q622^{def}	A1019^{cef}	L1716^{e}	A2077^{f}
**R61**^{cdef}	R188^{ef}	L627^{e}	**T1022**^{cdef}	W1717^{cef}	F2130^{e}
A62^{e}	T205^{ef}	R633^{f}	**R1023**^{cdef}	M1740^{ce}	W2142^{f}
C65^{de}	S206^{ef}	W636^{f}	Y1190^{e}	T1743^{e}	Y2189^{ef}
H74^{e}	**T209**^{cdef}	W637^{ef}	V1208^{f}	K1744^{e}	F2480^{f}
A76^{def}	L213^{f}	R663^{f}	D1211^{f}	K1784^{ef}	
F77^{e}	V233^{ef}	L778^{def}	F1249^{f}	Y1785^{ef}	
Q78^{f}	L235^{ef}	**R782**^{cdef}	Q1503^{f}	F1795^{f}	
C80^{ef}	A236^{e}	L786^{e}	F1504^{f}	F1932^{ef}	
H82^{f}	C261^{ef}	S789^{ef}	L1508^{ef}	R1935^{f}	

The simulated cholesterol concentration in which the residue exceeds cut-off is represented by superscript letters after each residue. Key: c – 10%, d – 20%, e – 30%, f – 40%. Residues which were above cut-off at all simulated cholesterol concentrations are in bold. Residues coinciding with CRAC or CARC motifs are shown in blue. Residues also exceeding cut-off in Piezo1$_{trunc}$ simulations are underlined. Residues located in the modelled N-terminal region are in an orange box.

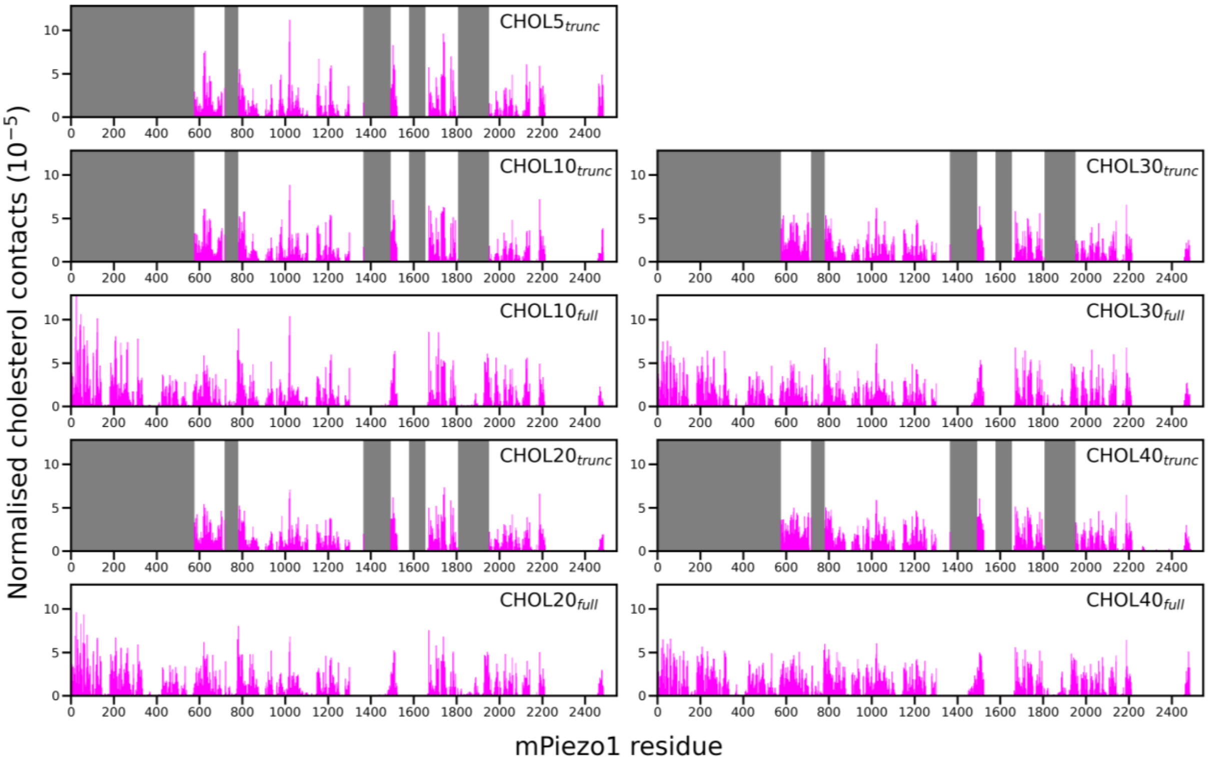

Supplementary Figure 5: Histograms of Piezo1-cholesterol contacts in all simulations. Each histogram is labelled with the corresponding simulation. Grey bars represent the residues missing from Piezo1$_{trunc}$.

Supplementary Table 4: CRAC and CARC motifs in mPiezo1

CRAC	CARC	
^{577}LVTGI**YV**K^{584}	^{113}KDIFNT**T**RLV^{122}	^{1674}RLLRAG**Y**QCV^{1683}
^{618}L**T**LF**Q**VYY**TL**WRK^{630}	^{290}RLFGLKNFV^{298}	^{1724}RPSK**R**FW**MT**AI**V**^{1735}
^{702}**LQ**L**HY**FHR^{709}	^{314}KHA**W**PI**Y**VSPGIL^{326}	^{1762}RYENKPYFPPRI**L**^{1774}
^{840}LPYPR**F**R^{846}	^{537}RQFVKEKLL^{545}	^{1778}KTDSYI**KY**DLV^{1788}
^{862}VCKMLYQLK^{870}	^{602}RLVVYKIV^{609}	^{1797}RSQLLCYGL^{1805}
^{935}V**F**EAVVYRR^{943}	^{629}RKLL**R**VF**WW**LVV^{640}	^{1941}RRL**Q**S**F**C**V**SL^{1950}
^{1058}LCID**Y**PWRWSK^{1068}	^{663}**R**NLTGFTDEQL^{673}	^{1960}RFFHDIL^{1966}
^{1076}LIKWLYLPDFFR^{1087}	^{709}RPFMQLTDL^{717}	^{1971}RAATDVYALMFL^{1982}
^{1252}VCTVKGYYDPK^{1262}	^{796}**RIQ**V**F**V**R**RLL^{805}	^{2035}RALYLRKTVL^{2044}
^{1602}LSTGYNTR^{1609}	^{811}**K**LVALYTVWVAL^{822}	^{2046}KLAFQVVLVV^{2055}
^{1758}VVLRRYENK^{1766}	^{864}KMLYQLKIV^{872}	^{2071}RMFSQN**A**V^{2078}
^{1776}LEKTDSYI**K**^{1784}	^{915}RKGYPNL^{921}	^{2086}KCIYFAL^{2092}
^{1950}LAQSF**Y**QPLQR^{1960}	^{979}KYFINFF**F**YKFGL^{991}	^{2098}RCGYPTRIL^{2106}
^{2032}VIDRALYLRK^{2041}	^{1005}RMNFMVIL^{1012}	^{2112}KKYNHLNL^{2119}
^{2078}VAQLWYFVK^{2086}	^{1031}RLWPNYCLFLTL^{1042}	^{2126}RLVP**F**LVEL^{2134}
^{2092}LSAYQIR^{2098}	^{1147}RSYLDMLKV^{1155}	^{2183}KKKIVK**Y**GMGGL^{2194}
^{2333}VEYTNEK^{2339}	^{1159}RYLFWLVLVV^{1168}	^{2231}KLGGYEPL^{2238}
^{2371}LFPKYIR^{2377}	^{1176}RISIFGLGYLL^{1186}	^{2318}RFTWNFQRDL^{2327}
^{2521}LEEELYAK^{2528}	^{1294}RRIFLSHYFL^{1303}	^{2422}KASDFLEWWV^{2431}
	^{1318}RGFALYNAANL^{1328}	^{2505}KLCQDIFLV^{2513}

Residue numbers are indicated. Residues with significant cholesterol interactions in any simulation are in bold and colored magenta.

Supplementary Table 5: Piezo1$_{trunc}$ residues with PIP$_2$ contacts exceeding cut-off of 70% of maximum contacts.

L577[ef]	<u>**R844**</u>[abcdef]	Q1496[e]
T579[acdef]	<u>**R846**</u>[abcdef]	**R1497**[abcdef]
**K584**[abcdef]	**R942**[abcdef]	K1727[abcde]
L627[cef]	R949[bcde]	R1728[abce]
**W628**[abcdef]	<u>**R1023**</u>[abcdef]	Q1952[b]
<u>**R629**</u>[abcdef]	<u>**R1024**</u>[abcdef]	R2040[acd]
<u>**K630**</u>[abcdef]	<u>**R1025**</u>[abcdef]	K2183[acde]
<u>**R633**</u>[abcdef]	**R1031**[abcdef]	K2184[abde]
H708[df]	<u>K1201</u>[c]	K2185[acde]
**R709**[abcdef]	<u>**R1204**</u>[abcdef]	G2188[c]
**R782**[abcdef]	**H1493**[abcdef]	R2517[cd]

The simulated cholesterol concentration of systems in which the residue exceeds the cutoff is indicated by superscript letters as follow: a – 0%, b – 5%, c – 10%, d – 20%, e – 30%, f – 40%. Residues which were above the cutoff in all the Piezo1$_{trunc}$ systems are in bold. Residues also exceeding cut-off in Piezo1$_{full}$ simulations are underlined.

Supplementary Table 6: Piezo1$_{full}$ residues with PIP_2 contacts exceeding cut-off of 70% of maximum contacts.

T56[ce]	**R466**[acdef]	**<u>R1024</u>**[acdef]	R1924[acd]
**G57**[acdef]	**<u>R629</u>**[acdef]	**<u>R1025</u>**[acdef]	H1925[ac]
**R58**[acdef]	**<u>K630</u>**[acdef]	<u>K1201</u>[ad]	**K1929**[acdef]
L60[ac]	**L632**[acdef]	**<u>R1204</u>**[acdef]	S1930[cd]
**R61**[acdef]	**<u>R633</u>**[acdef]	R1891[acdf]	K1931[acdf]
**R144**[acdef]	F711[cde]	R1892[acdf]	**R1933**[acdef]
R151[ef]	G724[ce]	R1893[acdf]	**R1935**[acdef]
**K186**[acdef]	**R726**[acdef]	K1894[cdf]	**K1937**[acdef]
**R187**[acdef]	**<u>R844</u>**[acdef]	R1920[acde]	R1941[acd]
R348[def]	<u>R846</u>[c]	**K1921**[acdef]	R1942[acdf]
K349[ef]	**<u>R1023</u>**[acdef]	R1922[cdf]	

The cholesterol concentration of systems in which the residue exceeds the cut-off is indicated by superscript letters as follow: a – 0%, c – 10%, d – 20%, e – 30%, f – 40%. Residues which were above the cut-off in all the Piezo1$_{full}$ systems are in bold. Residues also exceeding cut-off in Piezo1$_{trunc}$ simulations are underlined. Residues located in the modelled N-terminal region are in an orange box.

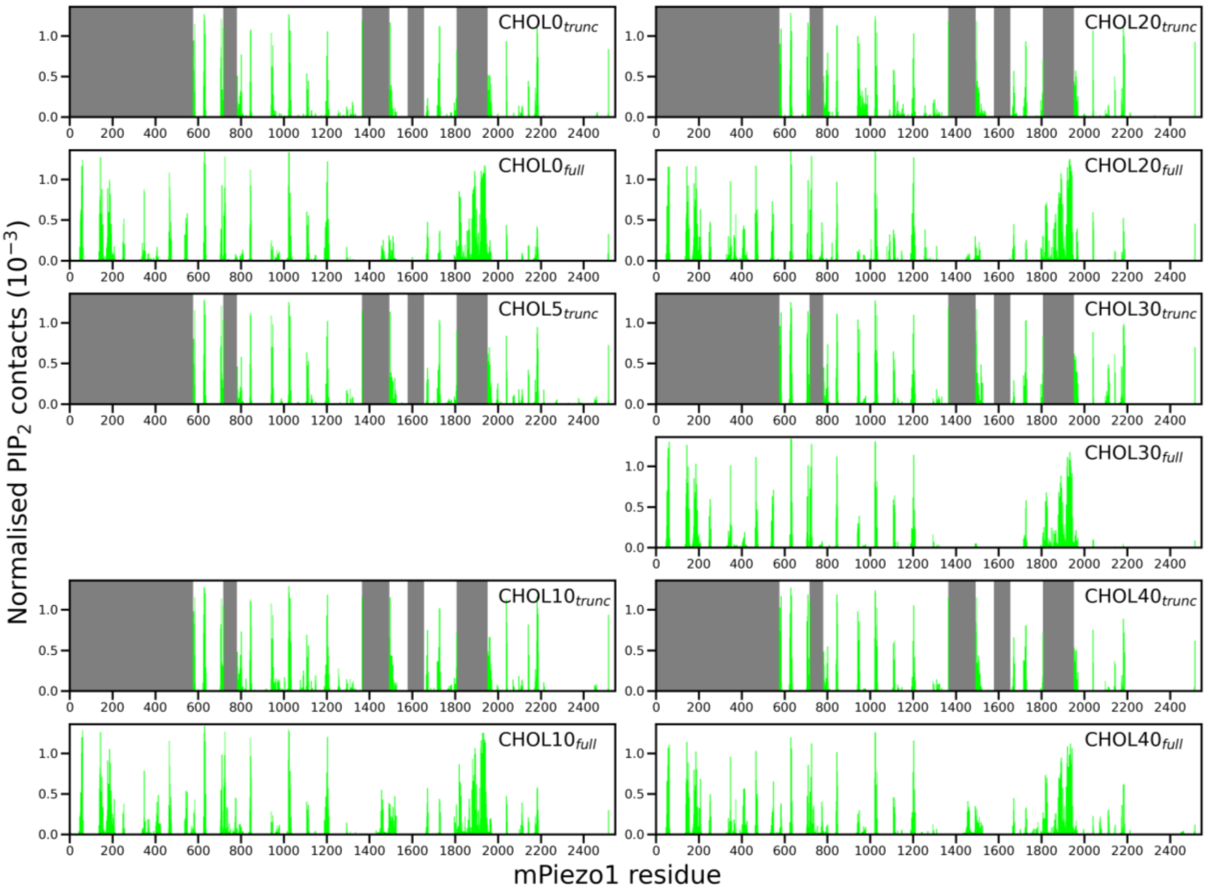

Supplementary Figure 6: Histograms of Piezo1-PIP$_2$ contacts in all simulations. Each histogram is labelled with the corresponding simulation. Grey bars represent the residues missing from Piezo1$_{trunc}$.

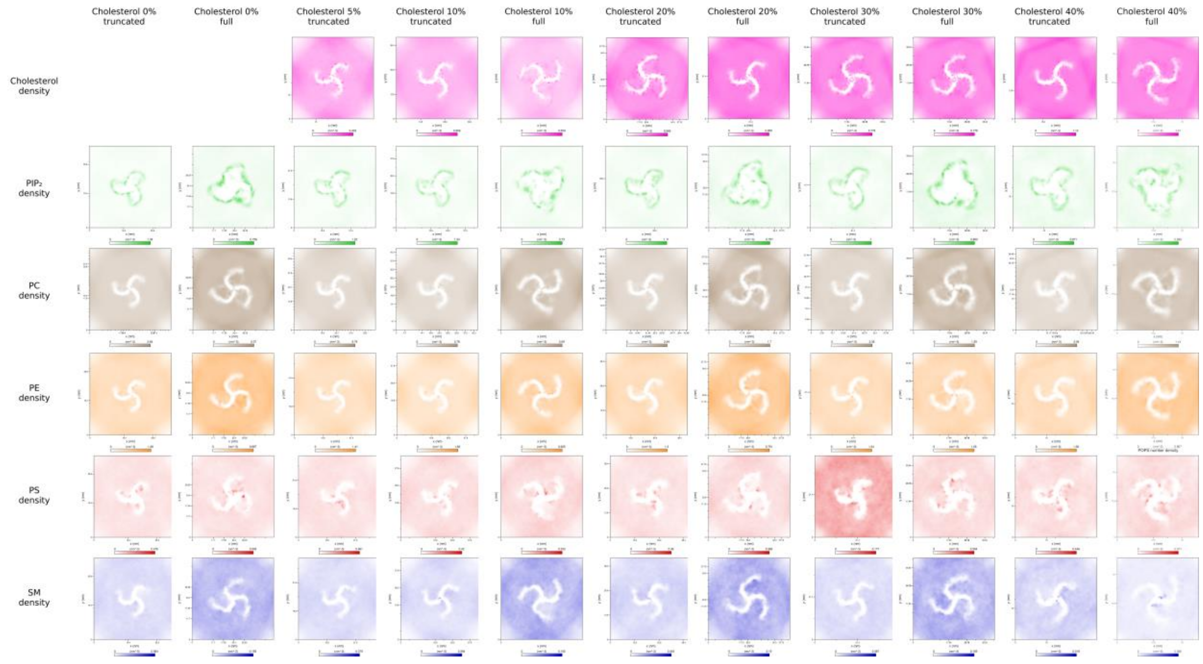

Supplementary Figure 7: Matrix of lipid density maps for all simulations conducted. Lipids are color coded as cholesterol – magenta, PIP_2 – green, POPC – tan, POPE – orange, POPS – red, SM – blue.

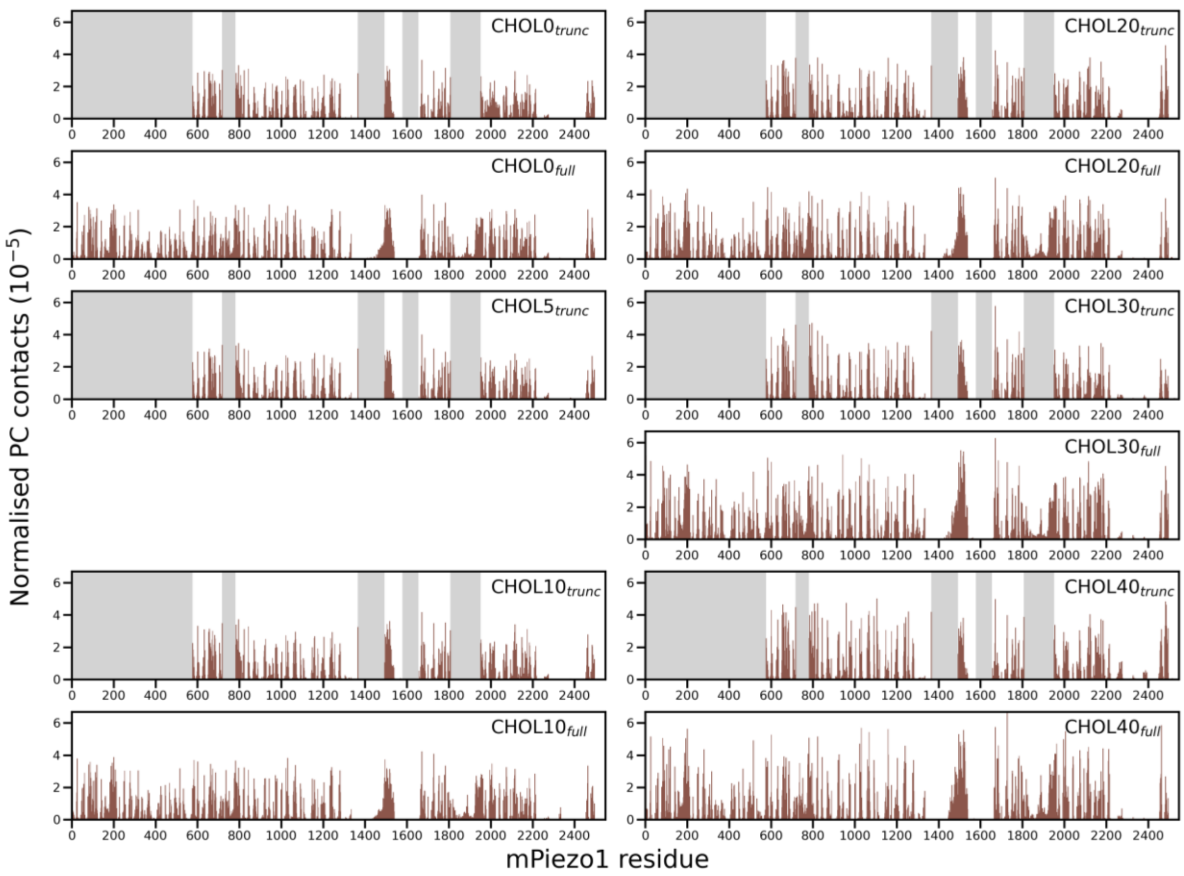

Supplementary Figure 8: Histograms of Piezo1-POPC contacts in all simulations. Each histogram is labelled with the corresponding simulation. Grey bars represent the residues missing from Piezo1$_{trunc}$.

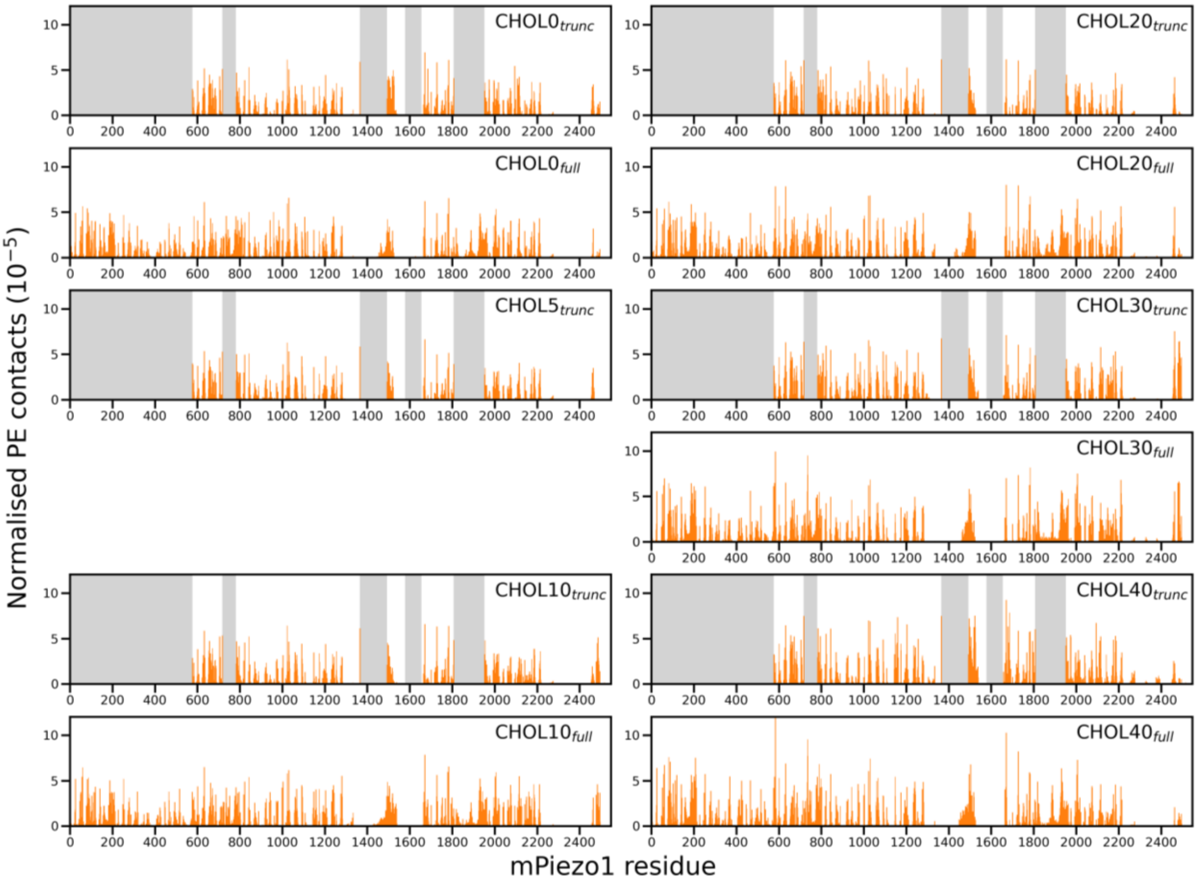

Supplementary Figure 9: Histograms of Piezo1-POPE contacts in all simulations. Each histogram is labelled with the corresponding simulation. Grey bars represent the residues missing from Piezo1$_{\text{trunc}}$.

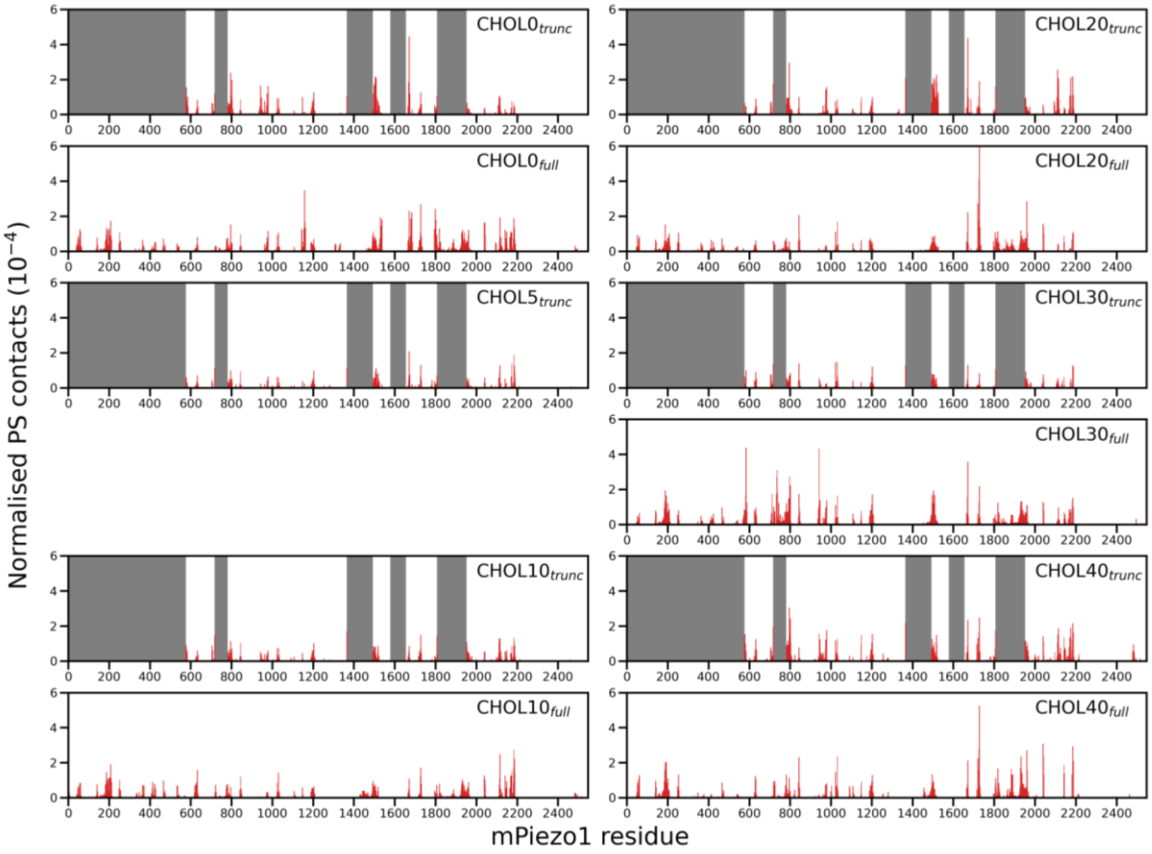

Supplementary Figure 10: Histograms of Piezo1-POPS contacts in all simulations. Each histogram is labelled with the corresponding simulation. Grey bars represent the residues missing from Piezo1$_{trunc}$.

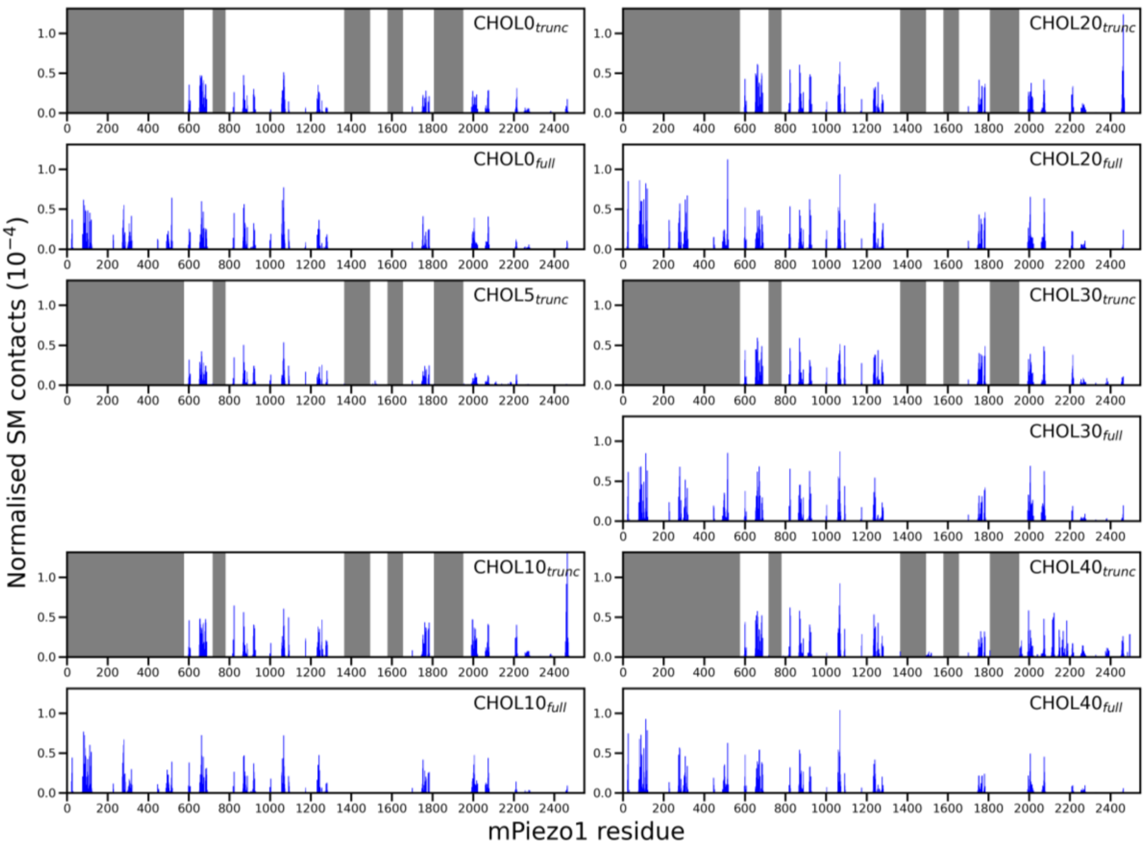

Supplementary Figure 11: Histograms of Piezo1-SM contacts in all simulations. Each histogram is labelled with the corresponding simulation. Grey bars represent the residues missing from Piezo1$_{trunc}$.
