# Supplementary material for: Modeling of full-length Piezo1 suggests importance of the proximal N-terminus for dome structure
Source: Biophys J. 2021 Feb 12;120(8):1343–56. doi: 10.1016/j.bpj.2021.02.003 (PMC8105715; doi:10.1016/j.bpj.2021.02.003)
Supplement: Document S2. Article plus supporting material [file mmc2.pdf]

Biophysical Journal
Article

Biophysical Society

Modeling of full-length Piezo1 suggests importance of the proximal N-terminus for dome structure

Jiehan Chong,[1] Dario De Vecchis,[1] Adam J. Hyman,[1] Oleksandr V. Povstyan,[1] Melanie J. Ludlow,[1] Jian Shi,[1] David J. Beech,[1,*] and Antreas C. Kalli[1,2,*]

[1]Leeds Institute of Cardiovascular and Metabolic Medicine, School of Medicine and [2]Astbury Centre for Structural Molecular Biology, University of Leeds, Leeds, United Kingdom

ABSTRACT Piezo1 forms a mechanically activated calcium-permeable nonselective cation channel that is functionally important in many cell types. Structural data exist for C-terminal regions, but we lack information about N-terminal regions and how the entire channel interacts with the lipid bilayer. Here, we use computational approaches to predict the three-dimensional structure of the full-length Piezo1 and simulate it in an asymmetric membrane. A number of novel insights are suggested by the model: 1) Piezo1 creates a trilobed dome in the membrane that extends beyond the radius of the protein, 2) Piezo1 changes the lipid environment in its vicinity via preferential interactions with cholesterol and phosphatidylinositol 4,5-bisphosphate (PIP_2) molecules, and 3) cholesterol changes the depth of the dome and PIP_2 binding preference. In vitro alteration of cholesterol concentration inhibits Piezo1 activity in a manner complementing some of our computational findings. The data suggest the importance of N-terminal regions of Piezo1 for dome structure and membrane cholesterol and PIP_2 interactions.

SIGNIFICANCE Piezo1 is a mechanosensitive ion channel with key roles in multiple systems and roles in at least two inherited disorders. How this channel carries out its roles is still unclear because we have only partial structural data and little or no knowledge of the structure in a lipid bilayer. In this study, we modeled the complete channel in an asymmetric membrane containing physiological lipids and conducted molecular simulations that suggest a trilobular membrane footprint for Piezo1 extending beyond the channel. The model also suggests that Piezo1 sequesters specific lipids and thereby alters the local lipid environment. These properties suggest cell type-specific roles of Piezo1 depending on cellular lipid composition and implications of Piezo1 for other proteins in its immediate vicinity.

INTRODUCTION

Piezo1 is a mechanosensitive ion channel found in many tissues (1). It plays key roles in the circulation (2–7), kidney (8), red blood cells (9,10), the immune system (11), the central nervous system (12), and other systems (1). These roles span the entire life cycle, from embryonic development (2–5) to maintenance of the mature organism (6–8) and ageing (12). Pathological mutations of Piezo1 lead to dehydrated hereditary stomatocytosis (DHS) through gain of function (13,14) and generalized lymphatic dysplasia through loss of function (15,16).

Piezo1 is inherently mechanosensitive (17–19), but how it senses force remains only partially understood. Piezo1 forms nonselective cationic channels with single channel conductance of ~29 pS for mouse Piezo1 (mPiezo1) (20). Structures of mPiezo1, excluding the N-terminal and some other regions, have been resolved by cryo-electron microscopy (cryo-EM) to 3.7–4.8 Å (21–24). These structures showed that Piezo1 adopts a trimeric configuration with a triskelion shape (21–24). The C-termini of the three Piezo1s converge to form a central ion pore, whereas the partially resolved N-terminal regions extend from the central axis, forming "blades" that spiral in plane with the cell membrane as they radiate outward, as well as curving toward the extracellular surface. On the intracellular surface, a helical beam connects the peripheral regions of the blades to the central regions (21). So far, no structure for full-length Piezo1 is available, thus limiting our ability to use structural data to understand Piezo1.

Submitted September 16, 2020, and accepted for publication February 3, 2021.

*Correspondence: d.j.beech@leeds.ac.uk or a.kalli@leeds.ac.uk

Jiehan Chong, Dario De Vecchis, and Adam J. Hyman first authors.

Editor: Alemayehu Gorfe.

https://doi.org/10.1016/j.bpj.2021.02.003

© 2021 Biophysical Society.

This is an open access article under the CC BY license (http://creativecommons.org/licenses/by/4.0/).

Ion channel function can be regulated by membrane lipids (25,26). In particular, cholesterol has the potential to affect the mechanogating of Piezo1 (27). The effect of cholesterol on other ion channel families is considerable but also varied. Cholesterol addition has been reported to increase the activity of Orai1 (28), Kv1.3 (29), Kir2 (30), and sodium currents in cardiac myocytes (31). Cholesterol depletion reduces the activity of ENaC (32) and TRPC (33) channels. Lateral organization of cholesterol in the membrane regulates Piezo1 activity (34), and indirect regulation occurs through STOML3, which tunes Piezo1 activity (35) as well as membrane stiffness (36). Cholesterol has previously been shown to regulate the activity of Ca^{2+}-permeable stretch-activated channels (37,38), but it is not known if these channels were Piezo1. It was also shown that some other mechanosensitive channels, e.g., MscL and MscS, have lipid pockets in their transmembrane (TM) region (39,40). Lipid acyl chains can exchange between these pockets with the membrane regulating the mechanical activation of these channels. Structural differences that are proximal to the lipid nanopockets of two MscL orthologs may result in differences in their functions (41). Additionally, phosphatidylinositol 4,5-bisphosphate (PIP_2) has been suggested to regulate Piezo1 function (42). Recent studies have also shown that molecules such as fatty acids, which alter the membrane structure, regulate Piezo1 activity (43,44).

A recent study has shown that the unique shape of Piezo1 induces a dome in the membrane, possibly creating an energy store that provides the basis for mechanogating (22). Using mechanical calculations in which the dome was modeled as hemispherical, it was suggested that membrane tension flattens the dome, providing energy that could open the Piezo1 channel. In addition to the membrane region curved by direct contact with Piezo1, it was also suggested that the local Piezo1-induced curvature generates a wider membrane footprint, which is hypothesized to amplify the sensitivity of Piezo1 to mechanical force (45). A study using atomic force microscopy showed that when activated, Piezo1 adopts a flatter configuration (46). The sensitivity of Piezo1 is tuned by membrane tension and stiffness, which may in turn be influenced by varying membrane lipid composition (36,44,47). Accordingly, a “force-from-lipids” model of mechanical activation has been proposed, in which nothing is required for Piezo1 mechanosensation other than the cell membrane and Piezo1 itself (36,47). Other proposed mechanisms for Piezo1 force sensing include shear force deflection of the extracellular cap domain, membrane thinning in response to stretch and changes to membrane lipid composition (20). In addition to mechanical force, the chemical agonist Yoda1, which appears to bind to Piezo1, can contribute to its activation (48).

Despite the recent functional and structural evidence about Piezo1, our understanding of how Piezo1 creates a unique footprint in the membrane and how lipids regulate Piezo1 function at the molecular level is still limited. This is partly because the structure of the full-length Piezo1 is not available and partly because critical molecular details about lipid interactions of the available structure are missing. In this study, to start to address these limitations, we built a three-dimensional (3D) full-length Piezo1 structural model ($Piezo1_{full}$) derived from the partial structure in closed conformation and inserted it in a model lipid bilayer. We used this model to explore potential molecular mechanisms. We conducted coarse-grained (CG) molecular dynamics (MD) simulations with increasing cholesterol concentrations for the truncated (partial) Piezo1 structure ($Piezo1_{trunc}$) and the constructed full-length Piezo1. The results suggest a complex trilobed membrane footprint for Piezo1, which varies in depth with cholesterol concentration. Additionally, we suggest Piezo1 interactions with PIP_2 lipids in these simulations, especially in the newly modeled N-terminal region, supporting the proposed role of PIP_2 lipids in enabling Piezo1 activation (42) and the importance of studies with the full-length protein.

MATERIALS AND METHODS

Cell culture

Human umbilical vein endothelial cells (HUVECs) were purchased from Lonza (Basel, Switzerland) and cultured in Endothelial Cell Basal Medium supplemented with 2% fetal calf serum (Sigma, St. Louis, MO) and the following growth factors: 10 ng ml^{-1} vascular endothelial growth factor, 5 ng ml^{-1} human basic fibroblast growth factor, 1 μg ml^{-1} hydrocortisone, 50 ng ml^{-1} gentamicin, 50 ng ml^{-1} amphotericin B, and 10 μg ml^{-1} heparin. These growth factors were supplied as a bullet kit (Cell Media and Bullet Kit; Lonza). HUVECs were passaged two to six times.

Human embryonic kidney (HEK) 293 cells stably expressing human Piezo1 (P1 HEK TREx) under a tetracycline-inducible promoter (7) or stably expressing mPiezo1 without need for tetracycline induction (49) were utilized. Cells used for Yoda1 experiments were cultured in Dulbecco’s modified Eagle’s medium-F12 GlutaMAX (Invitrogen, Paisley, UK). Cells used in patch-clamp experiments were cultured in Dulbecco’s modified Eagle’s medium (Invitrogen). All HEK 293 cell culture media were supplemented with 10% fetal calf serum (Sigma) and 1% penicillin/streptomycin (Sigma-Aldrich, St. Louis, MO). For patch-clamp experiments, the cells were plated on poly-lysine-coated coverslips in bath solution (see patch-clamp recording) at least 1 h before experiments.

All cells were maintained at 37°C in a humidified atmosphere containing 5% CO_2.

Short interfering RNAs

HUVECs were transfected at 90% confluence with 50 nM short interfering RNA (siRNA) using Lipofectamine 2000 in OptiMEM (Gibco, Waltham, MA), as per the manufacturer’s instructions (Invitrogen). Medium was replaced after 3–4 h, and cells were used for experimentation 48 h post-transfection. siRNA was provided from Ambion (Austin, TX). The sequence used for Piezo1 siRNA was GCCUCGUGGUCUACAAGAUtt. Scrambled negative control siRNA provided by Ambion was used as a control.

Intracellular Ca^{2+} measurement

Cells were incubated with fura-2AM (2 μM) (Thermo Fisher Scientific, Waltham, MA) for 60 min at 37°C followed by a 30-min wash at room

temperature. All cholesterol treatments were before the wash for 30 min at 37°C and maintained during Ca^{2+} measurements. Measurements were made at room temperature (21–23°C) on a 96-well plate reader (FlexStation; Molecular Devices, San Jose, CA). The change (Δ) in intracellular Ca^{2+} concentration was indicated as the ratio of fura-2 emission (510 nm) intensities for 340- and 380-nm excitation (F340/380). The recording solution (standard bath solution) contained (mM) as follows: 130 NaCl, 5 KCl, 8 D-glucose, 10 HEPES, 1.2 $MgCl_2$, and 1.5 $CaCl_2$, titrated to pH 7.4 with NaOH.

Cholesterol treatment

Cholesterol in complex with methyl-β-cyclodextrin (MβCD) (cholesterol at 40 mg g^{-1}) and MβCD were purchased from Sigma. Cells were incubated with cholesterol-enriching or -depleting agents for 30 min at 37°C in standard bath solution.

Yoda1

Yoda1 (2-[5-[[(2,6-Dichlorophenyl)methyl]thio]-1,3,4-thiadiazol-2-yl]pyrazine) from Tocris Bioscience (Bristol, UK) was prepared in stocks at 10 mM in dimethyl sulfoxide (Sigma).

Patch-clamp recording

Macroscopic membrane currents through outside-out patches were recorded using standard patch-clamp technique in voltage-clamp mode at room temperature (21–23°C). The holding voltage was −80 mV. Patch pipettes were fire polished and had a resistance of 4–7 MΩ when filled with pipette solution. The bath and pipette solutions were identical and contained (in mM) as follows: NaCl 140, HEPES 10, and EGTA 5 (pH 7.4 with NaOH). Recordings were made using an Axopatch 200B Amplifier (Axon Instruments, Bristol, UK) equipped with Digidata 1550B and pClamp 10.6 software (Molecular Devices). Pressure steps of 200-ms duration were applied to the patch pipette using a High-Speed Pressure Clamp HSPC-1 System (ALA Scientific Instruments, Farmingdale, NY). Current records were analog filtered at 1 kHz and digitally sampled at 5 kHz.

Statistical analysis of cell-based data

The study was aimed at discovering components of a biological mechanism using various cell/molecular studies to address a single hypothesis. In the absence of prior knowledge of the mechanism, power calculations were not considered to be applicable. We selected numbers of independent repeats of experiments based on prior experience of studies of this type. In all cases, the number of independent repeats was at least 4.

For Yoda1 experiments, OriginPro 9.1 was used for data analysis and graph production. Averaged data are presented as mean ± SE.

For patch-clamp experiments, data were analyzed and plotted using pClamp 10.6 and MicroCal Origin 2018 (OriginLab). Averaged data are presented as mean ± SE.

Data were produced in pairs (test and control), and these data pairs were compared statistically using *t*-tests. One-way ANOVA followed by Tukey post hoc test was used for comparing multiple groups. Statistically significant difference is indicated by * ($p < 0.05$) and no significant difference by NS ($p > 0.05$). The number of independent experiments is indicated by n.

Outlying data were not detected or excluded. The number of replicates per independent experiment was four for Ca^{2+} assays and one for each patch-clamp assay consisting of five different cell recordings over 2 separate days.

Molecular modeling of the $Piezo1_{trunc}$

Missing loops were modeled on the published mPiezo1 structure (Protein Data Bank, PDB: 6B3R) (22) using MODELER (v9.19) (50). Five models were generated, and the best model was selected using the discrete optimized protein energy (DOPE) method (51). Large intracellular loops remained unstructured after modeling and were removed from the final $Piezo1_{trunc}$ model before simulation. These loops were located at residues 718–781, 1366–1492, 1579–1654, and 1808–1951.

Molecular modeling of full-length Piezo1 structure

Structural data were obtained from the published cryo-EM structure (PDB: 6B3R). Missing residues were added with MODELER (v 9.19), and the loop refinement tool (52) was used to remove a knot in one chain between residues 2066–2074. The best model was selected out of 10 candidates according to the DOPE method. To model the missing three N-terminal bundles from the template 6B3R (i.e., residues 1–576), we first carried out a transmembrane and structural prediction using MEMSAT-SVM (53) and PSIPRED webserver (54,55). As a structural template, we used the bundles 4-5-6 from a Piezo1 blade (i.e., residues 577–1129) in combination with MODELER. The PSI/TM-Coffee web tool in slow/accurate homology extension mode was used to obtain the target-template alignment. The final target-template alignment was carefully checked and manually modified to avoid fragmentation of secondary structure elements and transmembrane helices. During modeling, we imposed canonical α-helix conformations for the residues 2–12, 97–103, and 183–189. The loop modeling routine of MODELER (52) was used to remove a knot between residues 149–182 and 294–317, selecting the best loop out of 5 according to the DOPE score. The position of the obtained bundles 1-2-3 with respect to the rest of the protein was obtained by superposing the bundle 3 to the bundle 4 from a single chain using UCSF Chimera (56). Subsequently, bundles 1-2-3 were manually moved to avoid superposition. This procedure ensured that the new modeled bundles 1-2-3 followed a similar direction of the partial Piezo1 blade resolved by cryo-EM. We then used UCSF Chimera to impose canonical α-helix conformation to cytoplasmic residues 567–587, 747–752, 776–806, 1420–1424, 1437–1446, 1449–1468, 1632–1635, 1645–1650, and 1926–1968 as predicted by PSIPRED. This full-length Piezo1 chain was superposed to the others in 6B3R using UCSF Chimera obtaining the trimeric Piezo1 structure. The Piezo1 full-length model obtained was energy minimized with GROMACS 5.0.7 (57) before simulations.

The N-terminal region of Piezo1 (residues 1–576) was submitted to the I-TASSER server. The models produced were aligned to the $Piezo1_{full}$ model and Piezo2 (PDB: 6KG7) using the Matchmaker function of Chimera 1.14 using α-carbon atoms with the Needleman-Wunsch alignment algorithm, the BLOSUM-62 substitution matrix, and an iteration cutoff of 2.

Coarse-grained simulations

The Piezo1 models obtained were converted to a coarse-grained resolution using the *martinize* script (58), and energy minimized in a vacuum with GROMACS 5.0.7. To model the protein secondary and tertiary structure, an elastic network model with a cutoff distance of 7 Å was used. The elastic force constant was 1000 kJ/mol. Other parameters in the coarse-grained conversion were set to *martinize* defaults. The CG-MD simulations were performed using the Martini 2.2 force field (58) and GROMACS 5.0.7. The elastic network restricts any major conformational change within the protein during the CG-MD simulations. For the equilibration simulation, Piezo1 models were inserted in a complex asymmetric bilayer using the INSert membrANE tool (59). The membrane compositions are described in Table S1. The system composition containing 20% cholesterol was chosen to mimic the physiological endothelial membrane (60–62). Other

cholesterol concentrations were generated based on this initial system. All systems were initially assembled in a simulation box of size 44 × 44 × 24 nm. The systems were neutralized with a 150 mM concentration of NaCl. The models were first energy minimized with emstep 0.001 nm and emtol 10 $\text{kJmol}^{-1}\text{nm}^{-1}$. The number of energy minimization steps was set at 5000, but the maximal force reached emtol before 5000 steps in all systems. Systems were then equilibrated with protein particles restrained (1000 $\text{kJmol}^{-1}\text{nm}^{-2}$) to allow the membrane bilayer to equilibrate around the model. Equilibration time was 500 ns for $\text{Piezo1}_{\text{full}}$ and 100 ns for $\text{Piezo1}_{\text{trunc}}$. These long CG-MD equilibration steps were essential to equilibrate the lipid bilayer around Piezo1 and reconstitute the dome previously hypothesized (22). All simulations were performed at 323 K, with protein, lipids, and solvent separately coupled to an external bath using the V-rescale thermostat (63) (coupling constant of 1.0). Pressure was maintained at 1 bar (coupling constant of 1.0) with semi-isotropic conditions and compressibility of 3×10^{-6} using the Berendsen barostat (64) during equilibrations and the Parrinello-Rahman barostat (65) during productions. Lennard-Jones and Coulombic interactions were shifted to zero between 9 and 12 Å and between 0 and 12 Å, respectively. After the equilibration phase of the Piezo1 full-length, we removed lipid molecules that flipped between leaflets to restore the membrane asymmetry. After this step, the systems were further energy minimized and, when applicable, neutralized with counterions. A preliminary run of 5 ns using an integration step of 10 fs was carried out before the production phase. For each Piezo1 model, five unrestrained repeat simulations of 3 μs each were run using an integration step of 20 fs.

Piezo1 footprint depth analysis

A Python script was written to measure the depth of the Piezo1 dome. First, the simulation trajectory is fitted to the protein coordinates using the GROMACS tool gmx trjconv. The coordinates of CG phosphate headgroup residues in each frame of the fitted trajectory are extracted by a Python script, which performs the following analysis. The phosphate atoms are then separated into bilayer leaflets using a branching network algorithm. Briefly, this method starts with a single particle and identifies the other headgroup particles that are within a cutoff distance (2 nm) of the starting residue. The cutoff distance is selected to be smaller than the separation between the bilayer leaflets. Headgroup particles identified in this way are added to the same leaflet as the starting residue. This process iterates repeatedly until no more new particles can be added, and the remaining residues are assumed to be part of the other leaflet. The process is then repeated starting in the other leaflet to confirm that the leaflet identification is correct.

For each leaflet, the depth of the dome is calculated as the difference between the surface level and the bottom of the dome. The surface level is taken to be the average z-coordinate for the headgroup residues with z-coordinate in excess of the 90th centile. The average is used here to minimize the effect of random fluctuation of the membrane. For the bottom of the dome, the z-coordinate of the headgroup residue with the absolute lowest z-coordinate is used. This is because the bottom of the dome is prone to far less fluctuation, being fixed to Piezo1, which in turn is the fitting reference for the rest of the simulation. For the error calculation, the values of the depth from the three systems were merged together, and the standard error of mean was calculated.

To generate the height map of the bilayer leaflets, for each frame, CG phosphate beads were binned along the x and y axes: 75 bins for each axis. The average z-coordinate of beads contained in each bin was calculated and stored in a matrix for each frame. The matrices of all frames are averaged to create the final height map, which is plotted using the Matplotlib library.

Code used is available at: https://github.com/jiehanchong/membrane-depth-analysis.

The first microsecond of simulation data is excluded from depth analysis as this time is required for the footprint depth to reach equilibrium.

Analysis of protein and lipid density

The repeat simulations for each system were concatenated and fitted to the Piezo1 pore (residues 2105–2547). gmx densmap from the GROMACS package was used to generate a two-dimensional density map for protein and individual lipid species, with summation of density along the z axis. For each lipid species, the density map was normalized according to the number of molecules for that lipid in the system, and the result was plotted using the Python Matplotlib library.

Analysis of protein-lipid contacts and lipid binding preference

All five repeat simulations were concatenated for each system. Contact between CG lipid headgroup beads and protein beads were calculated using gmx mindist from the GROMACS package. A cutoff distance of 0.55 nm was used to define a contact. The results were checked to ensure consistent results between each chain in the trimer. The contacts for individual Piezo1 chains were added together. Per-residue contact data were plotted using Grace (http://plasma-gate.weiz-mann.ac.il/Grace/). Lipid binding preference was calculated from whole-protein contact data using the following equation, based on that previously described by Ngo et al. (66):

$$\langle \delta_L \rangle = N_L - \langle N_A \rangle \left(\frac{n_L}{n_A} \right),$$

where N_L is the number of protein-lipid contacts for the lipid being evaluated, N_A is the total number of protein-lipid contacts for all lipids in the system, n_L is the number of molecules of the lipid being evaluated, and n_A is the total number of lipid molecules in the system.

The first 1.5 μs are excluded from analysis as PIP_2-Piezo1 contacts converge on equilibrium during this time. Ensemble average values for δ_L are displayed with standard error of mean for each system.

RESULTS

Piezo1 generates a trilobular dome and extensive penumbra in the membrane

A structural model of $\text{Piezo1}_{\text{trunc}}$ was generated by adding missing residues to the published structural data of the mouse protein (PDB: 6B3R; see Materials and methods and Fig. 1 *A*). The missing residues were integral in the C-terminal region and did not include the extensive N-terminal region missing from the structural data. We simulated $\text{Piezo1}_{\text{trunc}}$ using CG-MD simulations in a bilayer containing 20% cholesterol and a full complement of phospholipids and sphingomyelin ($\text{CHOL20}_{\text{trunc}}$ simulation; see Table S1). The headgroup composition of these bilayers mimics the physiological endothelial membrane (60–62). The simulation suggests that $\text{Piezo1}_{\text{trunc}}$ changes the curvature of the membrane in its immediate vicinity, creating a stable membrane dome (Fig. 1 *B*). This local deformation results in a wider membrane deformation with a complex 3D topology, which extends beyond the vicinity of $\text{Piezo1}_{\text{trunc}}$. This footprint creates elevated crests in the bilayer that radiate outward from the blade tips of $\text{Piezo1}_{\text{trunc}}$ (Fig. 1 *C*, in *blue*), whereas depressed valleys radiate from the regions between the arms (Fig. 1 *C*, in *white*). Therefore, $\text{Piezo1}_{\text{trunc}}$ creates a

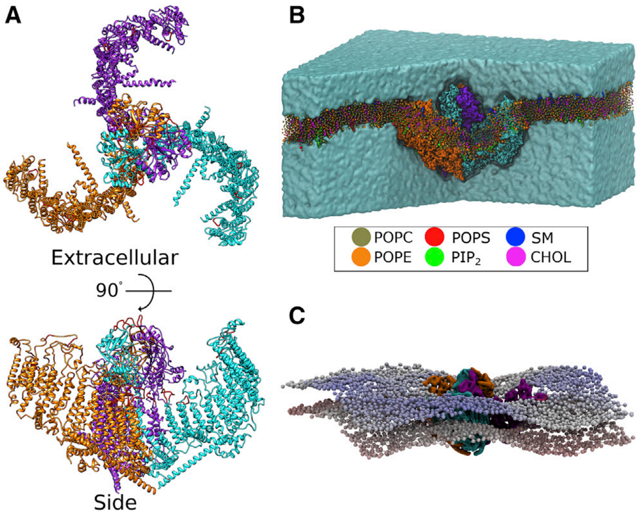

FIGURE 1 Piezo1 generates a complex membrane footprint. (*A*) Piezo1$_{trunc}$ model is shown from an extracellular and side view. Piezo1 chains are shown in cyan, orange, and purple. Modeled loops that were not present in the cryo-EM data are shown in red. (*B*) Shown is a snapshot from the end of the CHOL20$_{trunc}$ simulation. Some of the solvent and bilayer were removed to illustrate the curvature of the bilayer induced by the transmembrane regions of Piezo1. Legend indicates colors for POPC, phosphatidylethanolamine (POPE), phosphatidylserine (POPS), phosphatidylinositol-4,5-biphosphate (PIP_2), sphingomyelin (SM), and cholesterol (CHOL). (*C*) A snapshot from one of our CHOL20$_{trunc}$ simulation is shown. The Piezo1$_{trunc}$ backbone is displayed in surface representation. Phosphate beads are colored by their *z*-coordinates, from red at the bottom to blue at the top, illustrating crests and valleys in the bilayer. To see this figure in color, go online.

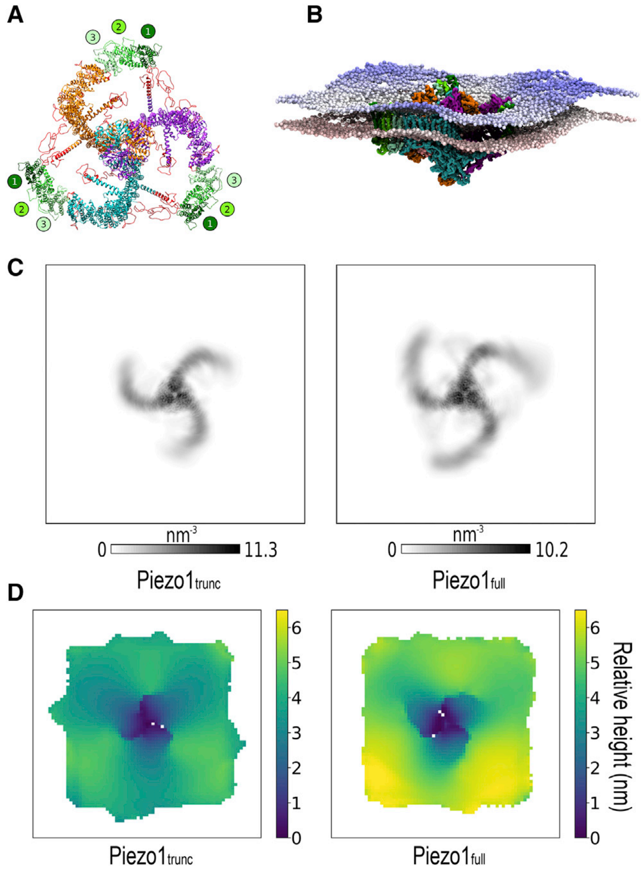

FIGURE 2 The full-length Piezo1 model has flexible distal N-terminal blades and enhances the footprint produced by the truncated mPiezo1 structure. (*A*) Extracellular view of the full-length mPiezo1 model. Chains are colored as in Fig. 1 *A*. The modeled transmembrane N-terminal bundles (residues 1–576) are numbered and colored from dark to light green. Other residues not resolved in the cryo-EM structure are colored red. (*B*) Final snapshot from one of the CHOL20$_{full}$ simulations. The Piezo1$_{full}$ backbone particles are displayed in surface representation. Phosphate beads are colored according to their *z*-coordinates from red at the bottom to blue at the top, illustrating the complex topology of the bilayer footprint. (*C*) Shown is the two-dimensional protein density of Piezo1$_{trunc}$ and Piezo1$_{full}$ during CHOL20$_{trunc}$ and CHOL20$_{full}$ simulations, respectively. Coordinates are fitted to the pore region (residues 2105–2547). (*D*) Shown are the height maps of CG phosphate beads corresponding to the extracellular leaflet in CHOL20$_{trunc}$ (*left*) and CHOL20$_{full}$ (*right*) simulations, averaged across all repeats. To see this figure in color, go online.

trilobed membrane topology that extends outside the radius of Piezo1$_{trunc}$.

The precise function and structure of the Piezo1 N-terminal residues 1–576 remains uncertain (21,22,24). However, the recent structure of the homologous Piezo2 (PDB: 6KG7) suggested that the presence of the missing N-terminal region will probably extend the Piezo1 transmembrane domain. After our Piezo1$_{trunc}$ simulations, we hypothesized that the structurally unresolved N-terminal residues might impact on the dimensions and dynamics of the Piezo1 footprint. Therefore, we built a full-length structural model for Piezo1. Using secondary structure prediction tools (see Materials and methods), we generated the topology for the N-terminal bundles. To build our model, we took advantage of the modular architecture of Piezo1 blades in which the first three 4-helical bundles have the same structure/topology with the adjacent group of three 4-helical bundles in Piezo1 blades, i.e., residues 577–1129 (bundles 4-5-6) of the resolved Piezo1 structure (PDB: 6B3R). Importantly, our model completes the Piezo1 structure by including cytoplasmic residues missing from the published structures (see Materials and methods). This provides the first 3D model for full-length Piezo1 (Piezo1$_{full}$) (Fig. 2 *A*). Our model is consistent with recent cryo-EM structure data for the homologous Piezo2 channel, in which the N-termini also continue to spiral and curve (67). Comparison of our Piezo1$_{full}$ model with the Piezo2 structure shows that the overall structures are very similar (Fig. S1 *E*). The N-terminal region of the blades of the Piezo2 structure is somewhat more elevated relative to our Piezo1$_{full}$ structure (Fig. S1 *F*); however, the Piezo1 blades are highly flexible in response to force (46), and the same is likely to be true of Piezo2. Therefore, differences between these static structures may not represent dynamic differences in vivo. We sought independent corroboration for our model by submitting the sequence of the modeled N-terminal residues (residues 1–576) to the independent modeling server I-TASSER (Fig. S2; (68,69)). The obtained models are of similar

topology with small differences mainly in the loop regions that connect the bundles.

In our Piezo1$_{full}$ model, each blade from a single chain is composed of 36 α-helices that form a total of nine 4-helix bundles as previously suggested (Fig. S1; (22)). As mentioned above, the Piezo1$_{full}$ model also comprises cytoplasmic residues not resolved by cryo-EM (Fig. 2 *A*, *red ribbons*; (22)). The assignment of loops to extracellular or intracellular positions agrees with previous experimental results, which used a combination of myc tagging and phosphorylation sites to distinguish extracellular and intracellular regions of Piezo1 (Fig. S1 *C*; (70)).

To assess how Piezo1$_{full}$ will alter the membrane footprint, CG-MD simulations were performed with the Piezo1$_{full}$ model inserted in a bilayer with identical lipid composition to CHOL20$_{trunc}$ (Table S1). In all five repeat simulations performed, the modeled N-terminal bundles remained embedded in the lipid bilayer (Fig. 2 *B*). To inspect the extent of blade movement over the course of the simulation, we analyzed the two-dimensional protein density for both Piezo1$_{trunc}$ and Piezo1$_{full}$ structural models (Fig. 2 *C*). We found that the protein density decreases in magnitude and becomes more diffuse, moving outward from the proximal C-terminal pore region toward the distal N-terminal blades. This effect is amplified in Piezo1$_{full}$, suggesting that the modeled N-terminal residues impart a wider range of movement to the blades, especially to the N-terminal region. This might explain the difficulties encountered in structural determination.

To analyze the topology of the Piezo1 footprint, we used a script that we developed (see Materials and methods) to generate an average height map of CG phosphate beads in each leaflet (Fig. 2 *D*). Surprisingly, this analysis reveals a footprint that is not uniformly circular but trilobular, as seen for the Piezo1$_{trunc}$. In comparison to Piezo1$_{trunc}$, Piezo1$_{full}$ produces a more pronounced footprint, with maximal depth exceeding 6 nm. In addition, the Piezo1$_{full}$ model produces a footprint with wider crests and narrower valleys. This result confirms the capacity of Piezo1 to indent the membrane and establishes a convoluted topology for the wider Piezo1 membrane footprint, to which the blades make an important contribution. Moreover, the deepest point of the dome is on the pore region. The footprint of Piezo1$_{trunc}$ appears to be somewhat more asymmetrical compared to that of Piezo1$_{full}$ (Fig. 2 *D*), with the relative height to be $\sim$6 nm on two sides and $\sim$5 nm in the third side.

To exclude bilayer asymmetry as a cause for the observed topology, we also simulated Piezo1$_{trunc}$ in a symmetric phosphatidylcholine (POPC) bilayer. This produced a similar bilayer topology (Fig. S3). The secondary and tertiary structure of Piezo1 is maintained during the simulations, with stable root mean-square deviation after $\sim$3 μs (Fig. S3 *B*). For the RMSD calculation, we have used the protein backbone particles. As expected, the RMSF of the core region is lower (between 0.2 and 0.5 nm) but rises toward the flexible N-terminal regions (0.5–1.2 nm) (Fig. S3 *C*).

The depth of the Piezo1 footprint varies with cholesterol concentration

It is suggested that the dimensions of the Piezo1 footprint may affect the sensitivity of Piezo1 to mechanical stress (45). Changes in the percentage of cholesterol are expected to affect the properties of the bilayer, particularly its curvature. To determine whether membrane cholesterol concentration influences the dimensions of the Piezo1 footprint, we performed further CG-MD simulations of Piezo1$_{trunc}$ and Piezo1$_{full}$ in a range of complex bilayers with cholesterol concentrations of 0, 5, 10, 30, and 40% (Table S1). For each cholesterol concentration, the depth of the Piezo1 footprint was analyzed using our script as above (see Materials and methods).

In the Piezo1$_{trunc}$ system, the deepest footprint is observed at 5% cholesterol, with a secondary peak at 30% cholesterol. The footprint is most shallow in between these peaks, at 10% cholesterol. In the Piezo1$_{full}$ system, the same reduction is observed between 0% and 10% but the depth of the dome remains almost similar as cholesterol concentration increases up to 40% (Fig. 3). These data show that the depth of the Piezo1 footprint varies nonlinearly with cholesterol concentration. They also suggest that the N-terminal bundles of Piezo1$_{full}$ make an important contribution to dome depth, not only by increasing the depth overall compared to the Piezo1$_{trunc}$ model but also by attenuating the effect of higher cholesterol concentrations on dome depth, which is seen with Piezo1$_{trunc}$.

Changing cholesterol concentration suppresses Piezo1 activation

To test the relevance of cholesterol changes to Piezo1 in vitro, we altered the cholesterol content of cells in culture. Treating modified HEK cells conditionally expressing human Piezo1 (P1 HEK TREx) with exogenous cholesterol reduced Ca^{2+} influx in response to Yoda1, a Piezo1 agonist (Fig. 4 *A*, *left*;

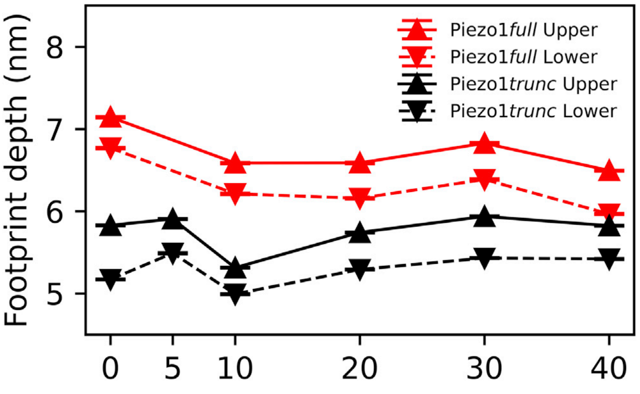

FIGURE 3 The depth of the Piezo1 membrane dome varies with cholesterol concentration. Average depth of the Piezo1 membrane dome in the CG-MD simulation with different membrane cholesterol concentrations. Systems and leaflets are indicated in the figure legend. To see this figure in color, go online.

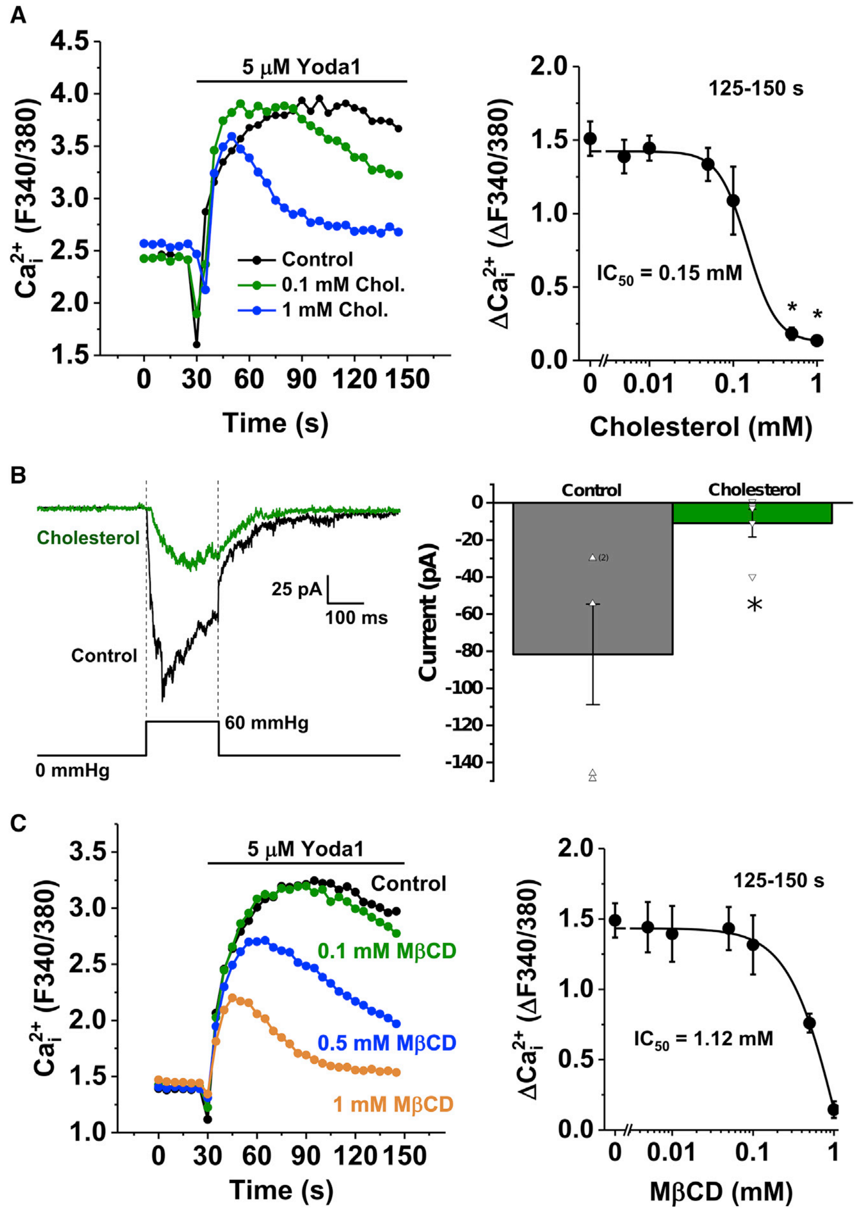

FIGURE 4 Modulation of Piezo1 by cholesterol. (*A*) Shown is the effect of exogenous cholesterol on Yoda1 responses in HEK cells conditionally expressing Piezo1 (P1 HEK TREx). Left, an example 96-well plate fura-2 measurement of the change in intracellular Ca^{2+} concentration evoked by 5 μM Yoda1 in cells pretreated with 0 (control), 0.1, and 1 mM cholesterol is shown. Right, mean data for the average amplitude (at 125–150 s) of intracellular Ca^{2+} responses to Yoda1 with varying doses of cholesterol (0.005–1 mM) displayed as a Hill equation, indicating the IC_{50} at 0.15 mM. n/N = 4/4. *p = <0.05. (*B*) Recordings were made using outside-out patch configuration applied to mouse P1 HEK TREx cells. Currents were evoked by 200-ms positive pressure steps of 60 mmHg applied to the patch pipette. The holding potential was −80 mV. Left, example current traces were recorded from two different patches preincubated with control solution (*black trace*) or solution containing 0.1 mM cholesterol applied for 30 min at room temperature before recording (*green trace*). Right, mean ± SEM of peak current for experiments of the type shown on the left, with all original data points superimposed (n = 5 for each group), is shown. (*C*) Effect of cholesterol depletion on Yoda1 responses in P1 HEK TREx is shown. Left, an example 96-well plate fura-2 measurement of the change in intracellular Ca^{2+} concentration evoked by 5 μM Yoda1 in cells pretreated with 0 (control), 0.1, 0.5, and 1 mM MβCD is shown. Right, mean data for the average amplitude (at 125–150 s) of intracellular Ca^{2+} responses with varying doses of MβCD (0.005–1 mM) displayed as a Hill equation are shown, indicating the IC_{50} at 1.12 mM. n/N = 4/4. To see this figure in color, go online.

(48)). The estimated 50% inhibitory effect (IC_{50}) of cholesterol was 0.15 mM (Fig. 4 *A*, *right*). Because of its limited solubility, higher concentrations of cholesterol were not tested. Mechanically activated mPiezo1, studied by electrophysiology in outside-out patches, was also inhibited by cholesterol (Fig. 4 *B*). We also sought to deplete membrane cholesterol using MβCD, a cholesterol-binding compound. Unexpectedly, MβCD also reduced the amplitude of Yoda1 responses in P1 HEK TREx (Fig. 4 *C*, *left*). The estimated IC_{50} was 1.12 mM (Fig. 4 *C*, *right*). The properties of Piezo1 can vary between overexpression and native environments (71), and so we also investigated HUVECs, which natively express Piezo1. In these cells, cholesterol and MβCD similarly, but more potently, inhibited Yoda1 responses (Fig. S4). α-Cyclodextrin (αCD), which is structurally similar to MβCD but does not bind cholesterol, had no effect (Fig. S4).

Together, these results suggest that an optimal cholesterol concentration for Piezo1 activity occurs at native membrane cholesterol levels in vitro, with either the addition or removal of cholesterol leading to a reduction in Piezo1 activity.

Piezo1 interacts with cholesterol in a model bilayer

To examine the interaction between cholesterol and Piezo1 in more detail, cholesterol density and protein-cholesterol contacts were analyzed in our CG-MD simulations of

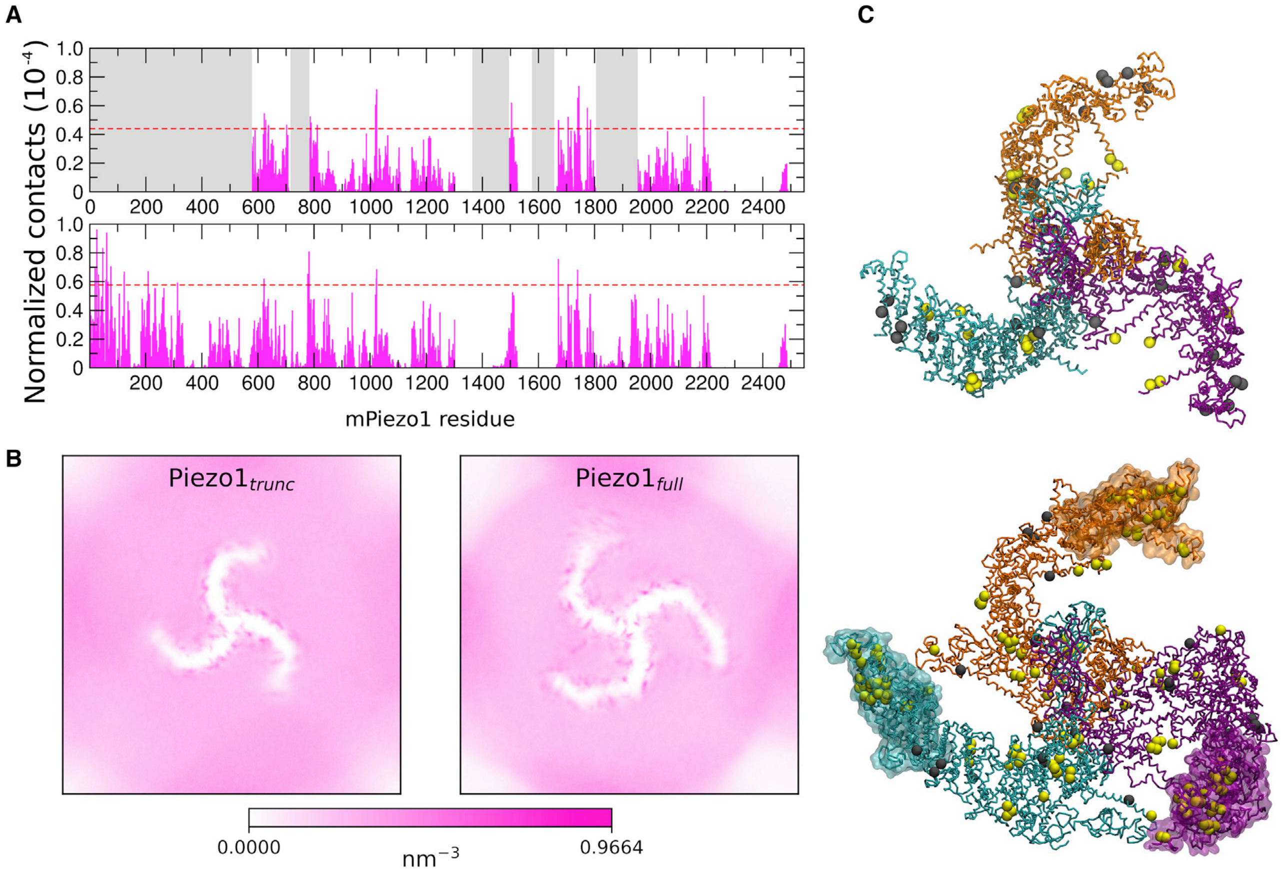

FIGURE 5 Piezo1 interactions with cholesterol in a complex model bilayer. (*A*) Shown are normalized contacts between cholesterol and Piezo1$_{trunc}$ (*top*) and Piezo1$_{full}$ (*bottom*) in the model bilayer containing 20% cholesterol. Normalization was done by dividing the number of cholesterol contacts of each residue by the number of cholesterol molecules and total number of frames analyzed. The grayed-out regions of the Piezo1$_{trunc}$ plot represent residues absent in Piezo1$_{trunc}$. Red dotted lines represent the cutoff of 60% of the contacts in each simulation. (*B*) Shown are two-dimensional density maps of cholesterol over five repeat simulations of CHOL20$_{trunc}$ and CHOL20$_{full}$, showing clustering of cholesterol along the Piezo1 blades. (*C*) Shown are snapshots of Piezo1$_{trunc}$ (*top*) and Piezo1$_{full}$ (*bottom*), with dynamic bonds between backbone residues colored as in Fig. 1 *A*. Cholesterol-interacting residues are displayed as yellow or gray spheres. Residues in yellow made significant interactions with cholesterol. Residues in gray both made significant interactions with cholesterol and are part of CRAC/CARC motifs. The modeled N-terminal regions of Piezo1$_{full}$ are displayed as transparent surfaces. To see this figure in color, go online.

Piezo1$_{trunc}$ and Piezo1$_{full}$. In our simulations, cholesterol clusters alongside structural regions, e.g., between the helices TM30 and TM31, TM22 and TM27, and adjacent to TM15 (Fig. 5, *A* and *B*). Using 60% of maximal contacts as a cutoff, 64 Piezo1$_{trunc}$ (Table S2) and 97 Piezo1$_{full}$ (Table S3) residues have significant contacts with cholesterol in one or more simulations. This regional clustering is observed across all cholesterol concentrations tested (Fig. S5). The N-terminal region of Piezo1, which is not present in the published structures, also makes significant interactions with cholesterol (Table S3).

CRAC and CARC consensus motifs are known cholesterol-binding domains (72). To determine the relevance of these motifs to Piezo1-cholesterol interactions, we searched for these motifs in the Piezo1 sequence (Table S4). 19 CRAC and 40 CARC motifs were identified. Of these, 8 CRAC and 15 CARC sequences overlap with residues with significant cholesterol interactions in either Piezo1$_{full}$ or Piezo1$_{trunc}$ (Fig. 5, *C*; Tables S3 and S4).

Predicted interaction of full-length Piezo1 with PIP$_2$

Lipid species other than cholesterol might also impact on Piezo1. For instance, PIP$_2$, a minority membrane lipid, is an important regulator of ion channels (73,74), including Piezo1 (42). Interestingly, in our analysis, PIP$_2$ interacts strongly with specific Piezo1 residues (Fig. 6 *A*). Across all simulations, there were 33 Piezo1$_{trunc}$ residues (Table S5) and 43 Piezo1$_{full}$ residues (Table S6) with contacts above the cutoff of 70% of the contacts in one or more systems. Of these, 17 are present in all Piezo1$_{trunc}$ systems (Table S5). 22 residues are over cutoff in all Piezo1$_{full}$ systems (Table S6). These include missing loops and bundles that have been modeled in Piezo1$_{full}$. 10 residues are above cutoff in both models: R629, K630, R633, R844, R846, R1023, R1024, R1025, K1201, and R1204. In simulations of both Piezo1$_{trunc}$ and Piezo1$_{full}$, PIP$_2$ lipids form an anionic annulus around Piezo1 in the inner leaflet (Fig. 6 *B*). Similar

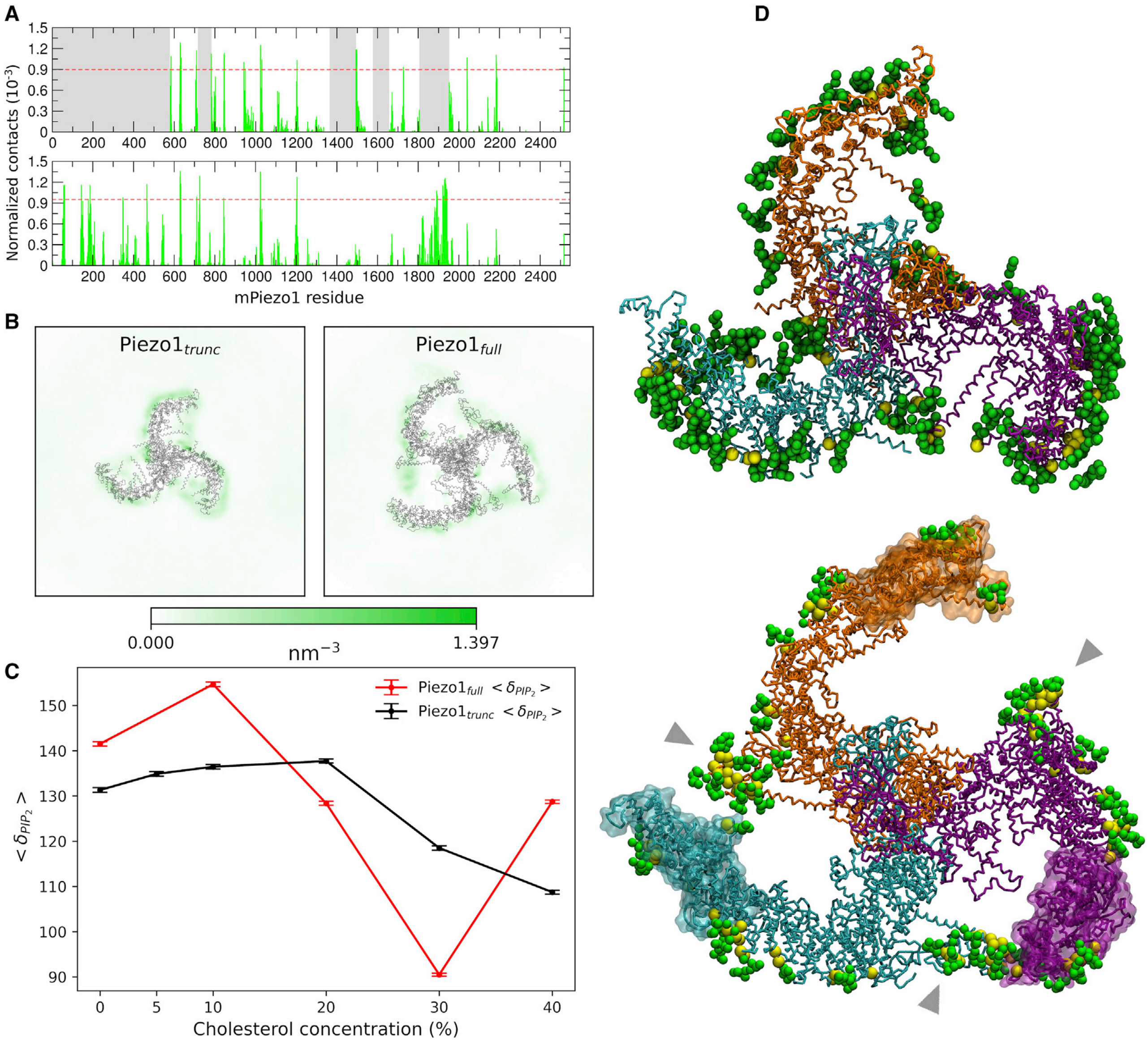

FIGURE 6 Piezo1 interactions with PIP_2 lipids. (*A*) Shown is the normalized number of contacts between PIP_2 and Piezo1$_{trunc}$ (*top*) and Piezo1$_{full}$ (*bottom*) in the CHOL20$_{trunc}$ and CHOL20$_{full}$. Grayed-out regions represent residues absent in Piezo1$_{trunc}$. Red dotted lines represent the cutoff of 70% of the contacts in each simulation. (*B*) Shown are normalized two-dimensional density maps of PIP_2 lipids in CHOL20$_{trunc}$ (*left*) and CHOL20$_{full}$ (*right*) with Piezo1 structures superimposed. (*C*) Shown is the thermodynamic preferential binding coefficient of PIP_2 relative to other lipid species (δ_{PIP2}) in Piezo1$_{full}$ and Piezo1$_{trunc}$ systems at cholesterol concentrations between 0 and 40%. (*D*) Shown are snapshots from one of our CHOL20$_{trunc}$ (*top*) or CHOL20$_{full}$ (*bottom*) simulations. The Piezo1 backbone is shown in the same colors as Fig. 1 *A*. Residues with PIP_2 contacts exceeding the cutoff of 70% maximal contacts are displayed as yellow spheres. PIP_2 lipids with headgroups within 10 Å of the Piezo1 residues that forms significant contacts with PIP_2 are displayed as green spheres. Gray arrows point to PIP_2 lipids clustering at the interface between adjacent Piezo1$_{full}$ blades. To see this figure in color, go online.

PIP_2 clustering (Fig. S6) and annulus formation (Fig. S7) is observed across all cholesterol concentrations tested, suggesting that PIP_2 binding site specificity is unaffected by cholesterol concentration. However, the thermodynamic preferential binding coefficient for PIP_2 does vary between cholesterol concentrations, with peak PIP_2 binding occurring at 10% cholesterol for the Piezo1$_{full}$ systems and between 10 and 20% cholesterol for the Piezo1$_{trunc}$ systems (Fig. 6 *C*).

The PIP_2 annulus around Piezo1$_{full}$ differs significantly from that around Piezo1$_{trunc}$. In Piezo1$_{full}$, the PIP_2 clustering occurs at the interface between the helices that lie in the plane of the inner leaflet and the convex surface of the adjacent blade (Fig. 6 *D*, *bottom*, *gray arrows*). There are regions of low PIP_2 density between the concavity of each blade and the central core of Piezo1, which may be a consequence of PIP_2 being drawn preferentially to the interfacial clusters (Fig. 6 *B*, *right*). These interactions of PIP_2

around Piezo1$_{full}$ are consistent across all cholesterol concentrations tested (Fig. S7) and suggest that the presence of the N-terminal region of Piezo1 changes this shape of the PIP_2 annulus around Piezo1.

Contact analysis of POPC (Fig. S8), phosphatidylethanolamine (Fig. S9), phosphatidylserine (Fig. S10), and sphingomyelin (Fig. S11) was also performed as well as density maps of all lipid species simulated (Fig. S7). We did not observe specific clustering or annulus formation for any lipid species other than PIP_2.

DISCUSSIONS

In this study, we model, for the first time to our knowledge, the full-length Piezo1 structure and its membrane footprint. The high mobility of the N-terminal residues observed in our simulations (Fig. 2) may explain why, to date, it has been challenging to resolve the structure of this region by cryo-EM (22–24). It has been suggested that in the inactive state, the Piezo1 N-terminal blades possibly flatten rather than continue to curve (22). However, our approach takes advantage of the modular topology that characterizes the Piezo fold and uses the existing solved transmembrane bundles as a template, leading to a continuous curved region toward the extracellular surface. This result is supported by the recently published cryo-EM structure for Piezo2, in which the N-termini also continue to curve (67).

Extension of the Piezo1 blades increases the depth of the Piezo1$_{full}$ footprint relative to Piezo1$_{trunc}$ (Fig. 2 *D*; Fig. 3). This observation couples Piezo1 blade conformation to local membrane curvature and supports the idea that the blades create, as well as sense, membrane curvature. Additionally, the high degree of mobility at the N-terminal ends of the blades may support a model in which Piezo1-induced curvature is dynamic and can change in response to force.

The Piezo1$_{full}$ model also includes extended unstructured loops on the cytoplasmic region that are also missing from the published structures (Fig. 2 *A*, *red ribbons*). Despite their lack of secondary structure, these loops contain numerous charged residues and may represent hotspots for cytoskeletal interactions or post-translational modification (e.g., phosphorylation), as has been previously suggested (70). There is evidence that cytoskeletal interaction is important in modulating mechanogating of Piezo1, with removal of the cytoskeleton leading to a decrease in the pressure required for channel opening (75).

Our results suggest that Piezo1 footprint has a trilobed topology with a dome protruding into the cytosolic membrane face, which extends beyond the immediate vicinity of the protein. Uniquely, our results also show that Piezo1 not only changes the membrane curvature around the protein, but it also changes its local lipid environment by creating an anionic annulus in the inner leaflet and by strong interactions with cholesterol. Inclusion of the N-terminal bundles in Piezo1$_{full}$ alters this membrane footprint and its relationship with cholesterol by increasing the dome depth, by changing the profile of its peaks and troughs, and by modifying its interactions with cholesterol and PIP_2. This may suggest that the N-terminal region plays a critical role in shaping the topology of the membrane surrounding Piezo1.

This Piezo1 footprint appears to be a long-range consequence of the immediate membrane indentation induced by Piezo1. In a report by Guo and Mackinnon (22), the depth of the dome created by Piezo1 is ~6 nm. This is consistent with the 6–7 nm depth seen in our simulations. We note that our results also suggest that the depth of the dome in the full-length Piezo1 is larger compared to the Piezo1$_{trunc}$, highlighting the role of the N-terminal region of the blades in regulating the Piezo1 membrane deformation. A wider perturbation of the membrane surrounding Piezo1 has also been suggested (45) using mechanical calculation and approximating the dome to a hemisphere. Our study shows that the Piezo1 footprint is far larger than Piezo1 radius and, for the first time to our knowledge, suggests that it has a uniquely complex trilobed topology with regions of varying curvature. This observation suggests that Piezo1 may be not only a passive receptor of mechanical forces but could transmit physical effects at long range through the membrane. This idea of Piezo1 applying force to the membrane while the membrane applies force to the protein is supported by recent work (46). The topology may also amplify Piezo1 tension sensitivity as previously suggested (22).

Membrane curvature is believed to be not just a consequence of cellular processes but also a generator of downstream effects, including membrane scission, fusion, protein sorting, and enzyme activation (76–79). The complex topology of the Piezo1 footprint, with regions of different height and curvature, raises intriguing possibilities regarding how Piezo1 could interact with membrane protein partners. Stable regions of differential curvature in the membrane might effectively function as coplanar compartments, with segregation of cellular processes by curvature. Membrane tension could be expected to alter the curvature difference between compartments, leading to alterations in protein aggregation (77) or lipid signaling (78). Hence, the complex Piezo1 footprint offers an alternative pathway for Piezo1-dependent mechanotransduction, beyond its properties as an ion channel. This possibility could be investigated using CG-MD simulation on a larger scale or experimentally with imaging techniques such as Förster resonance energy transfer (FRET) microscopy or stochastic optical reconstruction microscopy (STORM).

Our simulations suggest that membrane cholesterol composition regulates the dimensions of the dome created by Piezo1, with dome depth converging on a minimum at 10% cholesterol in the truncated version of Piezo1 and at 10 and 20% in the full-length model of Piezo1 and increasing at smaller or greater cholesterol concentrations. As these are CG simulations, the effect observed is likely

related to an effect on membrane stiffness impacting on dome depth rather than the effect of cholesterol binding at specific sites altering Piezo1 dynamics, as such effects would be poorly represented in a CG system. Our experimental results are in good agreement with this relationship because Piezo1 channel activity was at a peak in the native cholesterol HEK 293 cells or HUVECs, diminishing as cholesterol is added or removed. At this stage, it is unclear how the membrane cholesterol concentrations achieved in our in vitro work relate to the cholesterol concentrations used in our simulations. However, published data suggests that cholesterol forms around 20% of total HEK 293 cell lipids (80). Therefore, there are several potential explanations for the effect of altering cholesterol concentration on Piezo1 activity in vitro. One is a direct cholesterol effect, either because of the dome dimensions affecting the transmission of force through the membrane to Piezo1 or an effect of direct cholesterol binding on Piezo1 activity. Alternatively, the changes in PIP_2 binding affinity associated with alterations of membrane cholesterol concentration seen in our simulations could account for variation in Piezo1 activity, as depletion of PIP_2 (and its precursor phosphatidylinositol 4-phosphate) is associated with a reduction in Piezo1 activity (42). Given that neither footprint depth nor PIP_2 binding preference individually mirrors experimentally determined Piezo1 activity throughout the range of cholesterol concentrations tested, it may be that each mechanism contributes to a different extent at different cholesterol concentrations. Additionally, manipulation of membrane cholesterol content may disrupt lipid rafts, influencing the organization of Piezo1 and its interaction with partner proteins. Future studies could clarify these questions by testing the effects of cholesterol concentration on Piezo1 mutants that disrupt the predicted cholesterol and PIP_2 binding sites.

Our analysis suggests that some of the interactions of Piezo1 with cholesterol occur in CRAC and CARC motifs. It should be noted, however, that less than half of the CRAC or CARC on Piezo1 had significant cholesterol interactions. Although the interaction of CRAC and CARC motifs with cholesterol has been shown in a number of systems (72), their predictive value for cholesterol binding is limited because of the loose definition of the consensus sequence (81). Experimentally verifying the predictive value of these motifs would require mutating multiple stretches of amino acids in association with functional studies involving cholesterol. Such extensive changes could potentially have substantial structural effects, which would make it challenging to interpret results.

The ability of proteins to modify their local lipid environment has been described both computationally (82) and experimentally (83). Uniquely, in addition to its effect on local membrane curvature, Piezo1 changes its local lipid environment by forming an annulus of PIP_2 through strong preferential interaction. The preferential interaction of Piezo1 with lipids such as PIP lipids was also shown by other computational studies of the $Piezo1_{trunc}$ in complex model membranes (84). This is a demonstration of how a curved ion channel changes its local membrane environment both in terms of its lipid composition and of its topology. The PIP_2 annulus around Piezo1 could affect PIP_2 signaling by creating a hotspot around Piezo1 or acting as a PIP_2 sink to prevent PIP_2 signaling further away. This is especially so for the $Piezo1_{full}$ model, in which there is a high number of PIP_2 contacts with the modeled W1806-A1951 loop and N-terminal residues. Moreover, the annulus formed around $Piezo1_{full}$ has the additional feature of crossing the interface between blades of adjacent chains (Fig. 6 *D*, *gray arrows*) as well as relatively depleting PIP_2 around the Piezo1 core. The concentration of PIP_2 at the interface between Piezo1 blades may serve to stabilize their position, and relative paucity of PIP_2 around the core may tune PIP_2 sensitivity in this region to lower PIP_2 concentrations. One site which could be thus regulated is K2183. This site is homologous to K2167 on human Piezo1, which is the site of an in-frame deletion (human Piezo1 K2166-2169 deletion), which causes DHS (9). PIP_2 has been reported to regulate Piezo1 channel activity experimentally (42). Our data raise the possibility that this mutation may cause DHS through loss of PIP_2 regulation, but this remains to be tested in laboratory experiments.

The addition and removal of cholesterol reduced the activation of Piezo1 by Yoda1. In published work by Ridone et al. (34), mechanical activation of Piezo1 is similarly shown to be affected by the removal of cholesterol. In the same article, the addition of cholesterol does not appear to affect Piezo1 mechanoactivation, in contrast to our findings with Yoda1 activation. However, it should be noted that we required nearly 1 mM cholesterol concentration to see an effect on Piezo1 activation, whereas the maximal concentration of cholesterol-MβCD complex used in that article equates to a cholesterol concentration on the order of only 10 μM. Our findings are therefore compatible with this earlier work. The fact that cholesterol appears to affect both mechanical and Yoda1 activation, which occur by different mechanisms, suggests that these activation pathways may share a common component in which cholesterol is involved.

It might be possible that the common effect attributed to increase and reduction of cholesterol concentration could be due to the MβCD, which is used both to sequester cholesterol and in the form of cholesterol-MβCD complex to supplement cholesterol. This argument is partially addressed by our use of an αCD control. Substantial prior work has suggested that the main functional difference between these cyclodextrins is the property of β-cyclodextrin but not αCD to sequester cholesterol because of the different pocket sizes (85). They both sequester phospholipids, with αCD having the stronger effect (85), so sequestration of phospholipids is unlikely to explain the MβCD effect on Piezo1. One way to exclude a cholesterol-independent MβCD effect

would be to add MβCD to a cholesterol-free cell. Unfortunately, the complete absence of cholesterol had a severe adverse effect on cells, such that there was no recordable Piezo1 activity. A similar adverse reaction has been noted in prior work (85), presumably because of a fundamental requirement for cholesterol in cell function. Another approach would be to presaturate MβCD so that it cannot sequester cholesterol. In our case, the MβCD-cholesterol complex has an effect, which we have attributed to the cholesterol rather than the MβCD for the reasons above, and because MβCD is standard in the field for delivering cholesterol, which is otherwise insoluble. Thus, although we cannot completely exclude a cholesterol-independent MβCD effect, we have taken measures to minimize this possibility, which are in line with usual practice in this field.

Our computational work uses the closed curved structure of Piezo1. This is because it is the only state of Piezo1 for which we have an experimentally derived structure. Changes in the conformation of Piezo1 during activation may alter its lipid interactions. If more structural data become available for Piezo1, it will be possible to examine in more detail how changes in Piezo1 conformations may also change Piezo1/lipid interactions. The use of an elastic network model in our coarse-grained simulations that restrains Piezo1 in a closed curved conformation is another limitation. However, in this study, we wanted to study the Piezo1 membrane footprint in the absence of mechanical stimuli. The elastic network model achieves a reasonable approximation of this as without stimuli, Piezo1 is expected to remain in the closed curved state. Atomistic simulations with tension applied to the bilayer could provide more details on the conformational dynamics and activation of Piezo1.

CONCLUSIONS

This study proposes a 3D structure for the full-length Piezo1 channel, which has proven challenging to obtain experimentally. The full-length structure is critical when studying Piezo1 as it modifies the unique footprint of Piezo1 in a lipid bilayer. Piezo1 footprint has a trilobed topology that preferentially interacts with lipids such as PIP_2. Our computational and experimental results show that the Piezo1 footprint and function are modified by cholesterol, which could be important in atherosclerotic disease, a process in which both cholesterol and endothelial shear stress sensing, which Piezo1 contributes to, play a key role. Therefore, our findings suggest novel ways by which Piezo1 could act as an integrator of mechanical responses in health and disease.

SUPPORTING MATERIAL

Supporting material can be found online at https://doi.org/10.1016/j.bpj.2021.02.003.

AUTHOR CONTRIBUTIONS

J.C., D.D.V., A.J.H., J.S., D.J.B., and A.C.K. conceived the study. All authors were involved in experimental design. P1 HEK TREx cells were prepared by M.J.L. A.J.H. conducted calcium studies and associated data analysis. O.V.P. conducted electrophysiological studies and associated data analysis. J.C. conducted simulations of $Piezo1_{trunc}$, associated data analysis, and authored the membrane depth analysis script with input from A.C.K. D.D.V. built the $Piezo1_{full}$ model, conducted simulations of $Piezo1_{full}$, and associated data analysis.

ACKNOWLEDGMENTS

This research was funded by the Medical Research Council grant MR/R01745X/1 (J.C.), the Academy of Medical Sciences and the Wellcome Trust grant SBF002\1031 (D.D.V. and A.C.K.), the Biotechnology and Biological Sciences Research Council project 1360947 (A.J.H.), the Wellcome Trust grant 110044/Z/15/Z (O.V.P., M.J.L., and D.J.P.), and the British Heart Foundation grant FS/17/2/32559 (J.S.).

REFERENCES

1. Beech, D. J., and A. C. Kalli. 2019. Force sensing by Piezo channels in cardiovascular health and disease. *Arterioscler. Thromb. Vasc. Biol.* 39:2228–2239.
2. Li, J., B. Hou, ..., D. J. Beech. 2014. Piezo1 integration of vascular architecture with physiological force. *Nature.* 515:279–282.
3. Ranade, S. S., Z. Qiu, ..., A. Patapoutian. 2014. Piezo1, a mechanically activated ion channel, is required for vascular development in mice. *Proc. Natl. Acad. Sci. USA.* 111:10347–10352.
4. Nonomura, K., V. Lukacs, ..., A. Patapoutian. 2018. Mechanically activated ion channel PIEZO1 is required for lymphatic valve formation. *Proc. Natl. Acad. Sci. USA.* 115:12817–12822.
5. Choi, D., E. Park, ..., Y.-K. Hong. 2019. Piezo1 incorporates mechanical force signals into the genetic program that governs lymphatic valve development and maintenance. *JCI Insight.* 4:e125068.
6. Retailleau, K., F. Duprat, ..., E. Honoré. 2015. Piezo1 in smooth muscle cells is involved in hypertension-dependent arterial remodeling. *Cell Rep.* 13:1161–1171.
7. Rode, B., J. Shi, ..., D. J. Beech. 2017. Piezo1 channels sense whole body physical activity to reset cardiovascular homeostasis and enhance performance. *Nat. Commun.* 8:350.
8. Martins, J. R., D. Penton, ..., S. Demolombe. 2016. Piezo1-dependent regulation of urinary osmolarity. *Pflugers Arch.* 468:1197–1206.
9. Andolfo, I., S. L. Alper, ..., A. Iolascon. 2013. Multiple clinical forms of dehydrated hereditary stomatocytosis arise from mutations in PIEZO1. *Blood.* 121:3925–3935, S1–S12.
10. Cahalan, S. M., V. Lukacs, ..., A. Patapoutian. 2015. Piezo1 links mechanical forces to red blood cell volume. *eLife.* 4:e07370.
11. Solis, A. G., P. Bielecki, ..., R. A. Flavell. 2019. Mechanosensation of cyclical force by PIEZO1 is essential for innate immunity. *Nature.* 573:69–74.
12. Segel, M., B. Neumann, ..., K. J. Chalut. 2019. Niche stiffness underlies the ageing of central nervous system progenitor cells. *Nature.* 573:130–134.
13. Albuisson, J., S. E. Murthy, ..., A. Patapoutian. 2013. Dehydrated hereditary stomatocytosis linked to gain-of-function mutations in mechanically activated PIEZO1 ion channels. *Nat. Commun.* 4:1884.
14. Bae, C., R. Gnanasambandam, ..., P. A. Gottlieb. 2013. Xerocytosis is caused by mutations that alter the kinetics of the mechanosensitive channel PIEZO1. *Proc. Natl. Acad. Sci. USA.* 110:E1162–E1168.
15. Lukacs, V., J. Mathur, ..., B. L. Krock. 2015. Impaired PIEZO1 function in patients with a novel autosomal recessive congenital lymphatic dysplasia. *Nat. Commun.* 6:8329.

16. Fotiou, E., S. Martin-Almedina, …, P. Ostergaard. 2015. Novel mutations in PIEZO1 cause an autosomal recessive generalized lymphatic dysplasia with non-immune hydrops fetalis. *Nat. Commun.* 6:8085.
17. Syeda, R., M. N. Florendo, …, A. Patapoutian. 2016. Piezo1 channels are inherently mechanosensitive. *Cell Rep.* 17:1739–1746.
18. Wu, J., M. Young, …, J. Grandl. 2017. Inactivation of mechanically activated Piezo1 ion channels is determined by the C-terminal extracellular domain and the inner pore helix. *Cell Rep.* 21:2357–2366.
19. Wu, J., R. Goyal, and J. Grandl. 2016. Localized force application reveals mechanically sensitive domains of Piezo1. *Nat. Commun.* 7:12939.
20. Wu, J., A. H. Lewis, and J. Grandl. 2017. Touch, tension, and transduction - the function and regulation of Piezo ion channels. *Trends Biochem. Sci.* 42:57–71.
21. Ge, J., W. Li, …, M. Yang. 2015. Architecture of the mammalian mechanosensitive Piezo1 channel. *Nature.* 527:64–69.
22. Guo, Y. R., and R. MacKinnon. 2017. Structure-based membrane dome mechanism for Piezo mechanosensitivity. *eLife.* 6:e33660.
23. Saotome, K., S. E. Murthy, …, A. B. Ward. 2018. Structure of the mechanically activated ion channel Piezo1. *Nature.* 554:481–486.
24. Zhao, Q., H. Zhou, …, B. Xiao. 2018. Author correction: structure and mechanogating mechanism of the Piezo1 channel. *Nature.* 563:E19.
25. Rosenhouse-Dantsker, A., D. Mehta, and I. Levitan. 2012. Regulation of ion channels by membrane lipids. *Compr. Physiol.* 2:31–68.
26. Pliotas, C., and J. H. Naismith. 2017. Spectator no more, the role of the membrane in regulating ion channel function. *Curr. Opin. Struct. Biol.* 45:59–66.
27. Needham, D., and R. S. Nunn. 1990. Elastic deformation and failure of lipid bilayer membranes containing cholesterol. *Biophys. J.* 58:997–1009.
28. Derler, I., I. Jardin, …, C. Romanin. 2016. Cholesterol modulates Orai1 channel function. *Sci. Signal.* 9:ra10.
29. Hajdú, P., Z. Varga, …, R. Gáspár, Jr. 2003. Cholesterol modifies the gating of Kv1.3 in human T lymphocytes. *Pflugers Arch.* 445:674–682.
30. Romanenko, V. G., G. H. Rothblat, and I. Levitan. 2002. Modulation of endothelial inward-rectifier K+ current by optical isomers of cholesterol. *Biophys. J.* 83:3211–3222.
31. Wu, C.-C. C., M.-J. J. Su, …, Y.-T. T. Lee. 1995. The effect of hypercholesterolemia on the sodium inward currents in cardiac myocyte. *J. Mol. Cell. Cardiol.* 27:1263–1269.
32. Balut, C., P. Steels, …, D. Jans. 2006. Membrane cholesterol extraction decreases Na+ transport in A6 renal epithelia. *Am. J. Physiol. Cell Physiol.* 290:C87–C94.
33. Bergdahl, A., M. F. Gomez, …, K. Swärd. 2003. Cholesterol depletion impairs vascular reactivity to endothelin-1 by reducing store-operated Ca2+ entry dependent on TRPC1. *Circ. Res.* 93:839–847.
34. Ridone, P., E. Pandzic, …, B. Martinac. 2020. Disruption of membrane cholesterol organization impairs the activity of PIEZO1 channel clusters. *J. Gen. Physiol.* 152:604488.
35. Poole, K., R. Herget, …, G. R. Lewin. 2014. Tuning Piezo ion channels to detect molecular-scale movements relevant for fine touch. *Nat. Commun.* 5:3520.
36. Qi, Y., L. Andolfi, …, J. Hu. 2015. Membrane stiffening by STOML3 facilitates mechanosensation in sensory neurons. *Nat. Commun.* 6:8512.
37. Morachevskaya, E., A. Sudarikova, and Y. Negulyaev. 2007. Mechanosensitive channel activity and F-actin organization in cholesterol-depleted human leukaemia cells. *Cell Biol. Int.* 31:374–381.
38. Chubinskiy-Nadezhdin, V. I., Y. A. Negulyaev, and E. A. Morachevskaya. 2011. Cholesterol depletion-induced inhibition of stretch-activated channels is mediated via actin rearrangement. *Biochem. Biophys. Res. Commun.* 412:80–85.
39. Pliotas, C., A. C. E. Dahl, …, J. H. Naismith. 2015. The role of lipids in mechanosensation. *Nat. Struct. Mol. Biol.* 22:991–998.
40. Kapsalis, C., B. Wang, …, C. Pliotas. 2019. Allosteric activation of an ion channel triggered by modification of mechanosensitive nano-pockets. *Nat. Commun.* 10:4619.
41. Kapsalis, C., Y. Ma, …, C. Pliotas. 2020. In-lipid structure of pressure-sensitive domains hints Mechanosensitive channel functional diversity. *Biophys. J.* 119:448–459.
42. Borbiro, I., D. Badheka, and T. Rohacs. 2015. Activation of TRPV1 channels inhibits mechanosensitive Piezo channel activity by depleting membrane phosphoinositides. *Sci. Signal.* 8:ra15.
43. Conrard, L., A. Stommen, …, D. Tyteca. 2018. Spatial relationship and functional relevance of three lipid domain populations at the erythrocyte surface. *Cell. Physiol. Biochem.* 51:1544–1565.
44. Romero, L. O., A. E. Massey, …, V. Vásquez. 2019. Dietary fatty acids fine-tune Piezo1 mechanical response. *Nat. Commun.* 10:1200.
45. Haselwandter, C. A., and R. MacKinnon. 2018. Piezo's membrane footprint and its contribution to mechanosensitivity. *eLife.* 7:e41968.
46. Lin, Y. C., Y. R. Guo, …, S. Scheuring. 2019. Force-induced conformational changes in PIEZO1. *Nature.* 573:230–234.
47. Lewis, A. H. H., and J. Grandl. 2015. Mechanical sensitivity of Piezo1 ion channels can be tuned by cellular membrane tension. *eLife.* 4:e12088.
48. Syeda, R., J. Xu, …, A. Patapoutian. 2015. Chemical activation of the mechanotransduction channel Piezo1. *Elife.* 4:e07369.
49. Blythe, N. M., K. Muraki, …, N. A. Turner. 2019. Mechanically activated Piezo1 channels of cardiac fibroblasts stimulate p38 mitogen-activated protein kinase activity and interleukin-6 secretion. *J. Biol. Chem.* 294:17395–17408.
50. Šali, A., and T. L. L. Blundell. 1993. Comparative protein modelling by satisfaction of spatial restraints. *J. Mol. Biol.* 234:779–815.
51. Shen, M. Y., and A. Sali. 2006. Statistical potential for assessment and prediction of protein structures. *Protein Sci.* 15:2507–2524.
52. Fiser, A., R. K. G. K. Do, and A. Šali. 2000. Modeling of loops in protein structures. *Protein Sci.* 9:1753–1773.
53. Nugent, T., and D. T. Jones. 2012. Detecting pore-lining regions in transmembrane protein sequences. *BMC Bioinformatics.* 13:169.
54. Buchan, D. W. A., and D. T. Jones. 2019. The PSIPRED protein analysis workbench: 20 years on. *Nucleic Acids Res.* 47:W402–W407.
55. Jones, D. T. 1999. Protein secondary structure prediction based on position-specific scoring matrices. *J. Mol. Biol.* 292:195–202.
56. Pettersen, E. F., T. D. Goddard, …, T. E. Ferrin. 2004. UCSF Chimera–a visualization system for exploratory research and analysis. *J. Comput. Chem.* 25:1605–1612.
57. Abraham, M. J., T. Murtola, …, E. Lindahl. 2015. GROMACS: high performance molecular simulations through multi-level parallelism from laptops to supercomputers. *SoftwareX.* 1–2:19–25.
58. de Jong, D. H., G. Singh, …, S. J. Marrink. 2013. Improved parameters for the martini coarse-grained protein force field. *J. Chem. Theory Comput.* 9:687–697.
59. Wassenaar, T. A., H. I. Ingólfsson, …, S. J. Marrink. 2015. Computational lipidomics with *insane*: a versatile tool for generating custom membranes for molecular simulations. *J. Chem. Theory Comput.* 11:2144–2155.
60. Medow, M. S., L. Intrieri, …, M. E. Gerritsen. 1989. Dexamethasone effects on microvascular endothelial cell lipid composition. *Am. J. Physiol.* 257:C512–C519.
61. Takamura, H., H. Kasai, …, M. Kito. 1990. Phospholipid molecular species in human umbilical artery and vein endothelial cells. *J. Lipid Res.* 31:709–717.
62. Murphy, E. J., L. Joseph, …, L. A. Horrocks. 1992. Phospholipid composition of cultured human endothelial cells. *Lipids.* 27:150–153.
63. Bussi, G., D. Donadio, and M. Parrinello. 2007. Canonical sampling through velocity rescaling. *J. Chem. Phys.* 126:014101.
64. Berendsen, H. J. C. C., J. P. M. M. Postma, …, J. R. Haak. 1984. Molecular dynamics with coupling to an external bath. *J. Chem. Phys.* 81:3684–3690.

65. Parrinello, M., and A. Rahman. 1981. Polymorphic transitions in single crystals: a new molecular dynamics method. *J. Appl. Phys.* 52:7182–7190.

66. Ngo, V. A., S. Sarkar, …, A. E. Garcia. 2020. How anionic lipids affect spatiotemporal properties of KRAS4B on model membranes. *J. Phys. Chem. B.* 124:5434–5453.

67. Wang, L., H. Zhou, …, B. Xiao. 2019. Structure and mechanogating of the mammalian tactile channel PIEZO2. *Nature.* 573:225–229.

68. Yang, J., and Y. Zhang. 2015. I-TASSER server: new development for protein structure and function predictions. *Nucleic Acids Res.* 43:W174–W181.

69. Zhang, C., P. L. Freddolino, and Y. Zhang. 2017. COFACTOR: improved protein function prediction by combining structure, sequence and protein-protein interaction information. *Nucleic Acids Res.* 45:W291–W299.

70. Coste, B., S. E. Murthy, …, A. Patapoutian. 2015. Piezo1 ion channel pore properties are dictated by C-terminal region. *Nat. Commun.* 6:7223.

71. Evans, E. L., O. V. Povstyan, …, D. J. Beech. 2020. RBCs prevent rapid PIEZO1 inactivation and expose slow deactivation as a mechanism of dehydrated hereditary stomatocytosis. *Blood.* 136:140–144.

72. Fantini, J., and F. J. Barrantes. 2013. How cholesterol interacts with membrane proteins: an exploration of cholesterol-binding sites including CRAC, CARC, and tilted domains. *Front. Physiol.* 4:31.

73. Huang, C.-L. 2007. Complex roles of PIP_2 in the regulation of ion channels and transporters. *Am. J. Physiol. Renal Physiol.* 293:F1761–F1765.

74. Suh, B.-C., and B. Hille. 2008. PIP_2 is a necessary cofactor for ion channel function: how and why? *Annu. Rev. Biophys.* 37:175–195.

75. Cox, C. D., C. Bae, …, B. Martinac. 2016. Removal of the mechanoprotective influence of the cytoskeleton reveals PIEZO1 is gated by bilayer tension. *Nat. Commun.* 7:10366.

76. McMahon, H. T., and J. L. Gallop. 2005. Membrane curvature and mechanisms of dynamic cell membrane remodelling. *Nature.* 438:590–596.

77. Reynwar, B. J., G. Illya, …, M. Deserno. 2007. Aggregation and vesiculation of membrane proteins by curvature-mediated interactions. *Nature.* 447:461–464.

78. Chang-Ileto, B., S. G. Frere, …, G. Di Paolo. 2011. Synaptojanin 1-mediated PI(4,5)P2 hydrolysis is modulated by membrane curvature and facilitates membrane fission. *Dev. Cell.* 20:206–218.

79. McMahon, H. T., and E. Boucrot. 2015. Membrane curvature at a glance. *J. Cell Sci.* 128:1065–1070.

80. Dawaliby, R., C. Trubbia, …, C. Govaerts. 2016. Phosphatidylethanolamine is a key regulator of membrane fluidity in eukaryotic cells. *J. Biol. Chem.* 291:3658–3667.

81. Palmer, M. 2004. Cholesterol and the activity of bacterial toxins. *FEMS Microbiol. Lett.* 238:281–289.

82. Corradi, V., E. Mendez-Villuendas, …, D. P. Tieleman. 2018. Lipid-protein interactions are unique fingerprints for membrane proteins. *ACS Cent. Sci.* 4:709–717.

83. Prabudiansyah, I., I. Kusters, …, A. J. M. Driessen. 2015. Characterization of the annular lipid shell of the Sec translocon. *Biochim. Biophys. Acta.* 1848:2050–2056.

84. Buyan, A., C. D. Cox, …, B. Corry. 2020. Piezo1 forms specific, functionally important interactions with phosphoinositides and cholesterol. *Biophys. J.* 119:1683–1697.

85. Zidovetzki, R., and I. Levitan. 2007. Use of cyclodextrins to manipulate plasma membrane cholesterol content: evidence, misconceptions and control strategies. *Biochim. Biophys. Acta.* 1768:1311–1324.

Biophysical Journal, Volume *120*

Supplemental information

Modeling of full-length Piezo1 suggests importance of the proximal N-terminus for dome structure

Jiehan Chong, Dario De Vecchis, Adam J. Hyman, Oleksandr V. Povstyan, Melanie J. Ludlow, Jian Shi, David J. Beech, and Antreas C. Kalli

Supplementary Table 1: Membrane lipid compositions used for the CG-MD simulations

**Name**	**Extracellular leaflet**				**Cytoplasmic leaflet**				
	POPC	POPE	SM	CHOL	POPC	POPE	POPS	PIP2	CHOL
$CHOL0_{trunc}$	75	20	5	0	70	20	5	5	0
$CHOL5_{trunc}$	70	20	5	5	65	20	5	5	5
$CHOL10_{trunc}$	65	20	5	10	60	20	5	5	10
$CHOL20_{trunc}$	55	20	5	20	50	20	5	5	20
$CHOL30_{trunc}$	45	20	5	30	40	20	5	5	30
$CHOL40_{trunc}$	35	20	5	40	30	20	5	5	40
$CHOL0_{full}$	75	20	5	0	70	20	5	5	0
$CHOL10_{full}$	65	20	5	10	60	20	5	5	10
$CHOL20_{full}$	55	20	5	20	50	20	5	5	20
$CHOL30_{full}$	45	20	5	30	40	20	5	5	30
$CHOL40_{full}$	35	20	5	40	30	20	5	5	40

Lipid composition is expressed in molar percentages

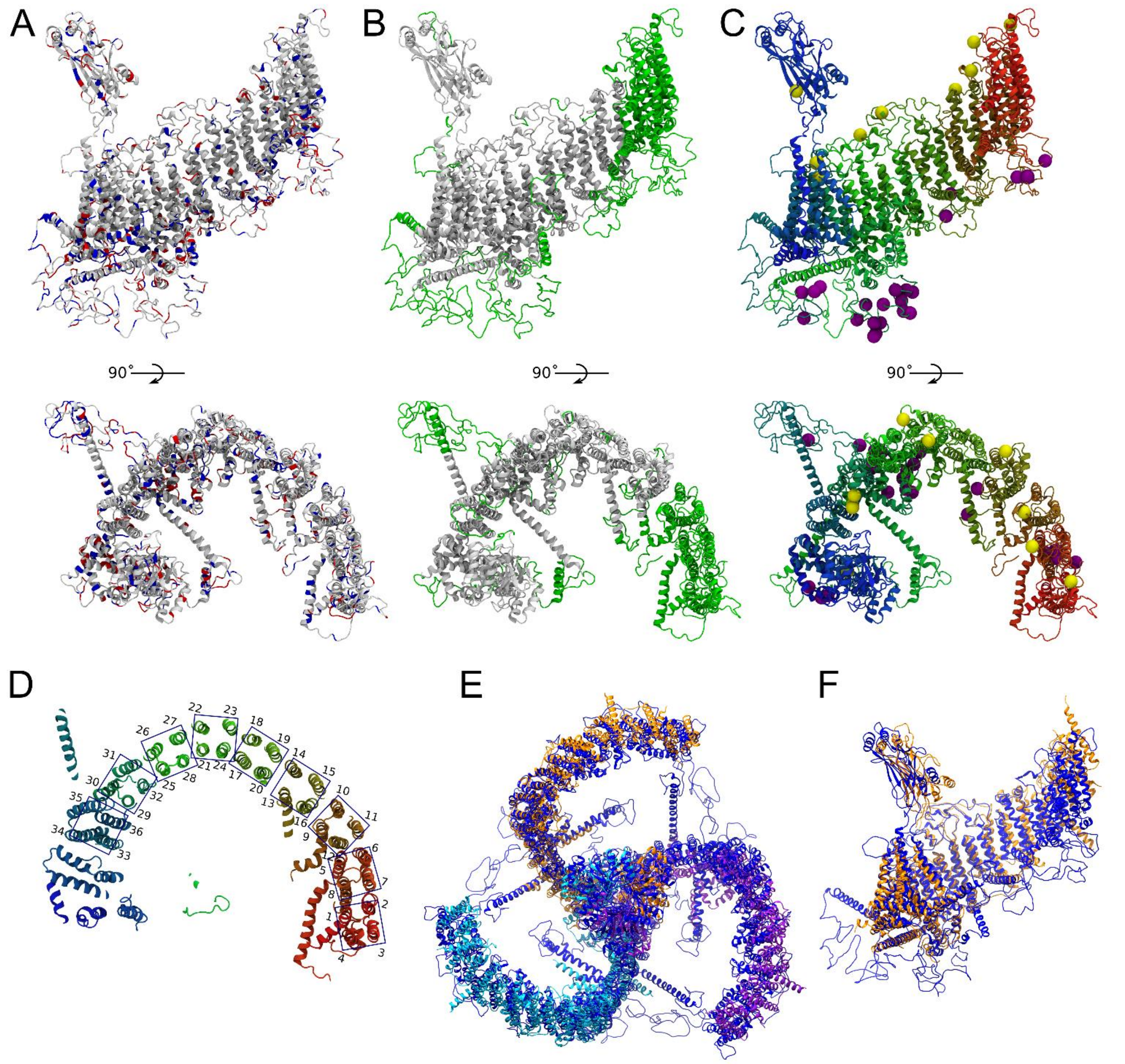

Supplementary Figure 1: Characterization of a single full-length chain Piezo1 mouse model. (A) A single full-length chain is shown as ribbon with positively (His, Lys, Arg) and negatively (Asp, Glu) charged residues colored in blue and red, respectively. **(B)** A single full-length chain is shown as ribbon with modelled missing residues from the PDB 6B3R, highlighted in green. **(C)** A single full-length chain is shown as ribbon and colored rainbow from N-terminal (red) to C-terminal (blue). Spheres are Cα atoms from residues previously identified as exposed to the extracellular side (Myc tagged, yellow) and phosphorylated (purple). Myc tagged residues are: 102, 304, 508, 669, 897, 1071, 1765, 2071, 2075 and 2336. Phosphorylated residues are: 351, 396, 397, 399, 738, 758, 1385, 1389, 1390, 1593, 1600, 1604, 1608, 1610, 1612, 1617, 1626, 1627, 1631, 1640, 1644, 1646 and 1837. **(D)** Cropped visualization of a single Piezo1 full-length chain. The numbering and position of each of the 36 bundles is indicated. **(E)** Structural superposition between the Piezo1 full-length model (blue) and the Piezo2 cryo-EM structure (chains are in orange, purple and cyan; PDB: 6KG7) performed with UCSF Chimera over the Cα atoms. **(F)** A single chain from the Piezo2 cryo-EM structure (orange) and a Piezo1 full-length model superposed with UCSF Chimera over their Cα atoms. The long loops in the cytoplasm (residues 718-781, 1366-1492, 1579-1654, 1808-1951, 542-568, 337-418, 145-182) were not considered in this alignment.

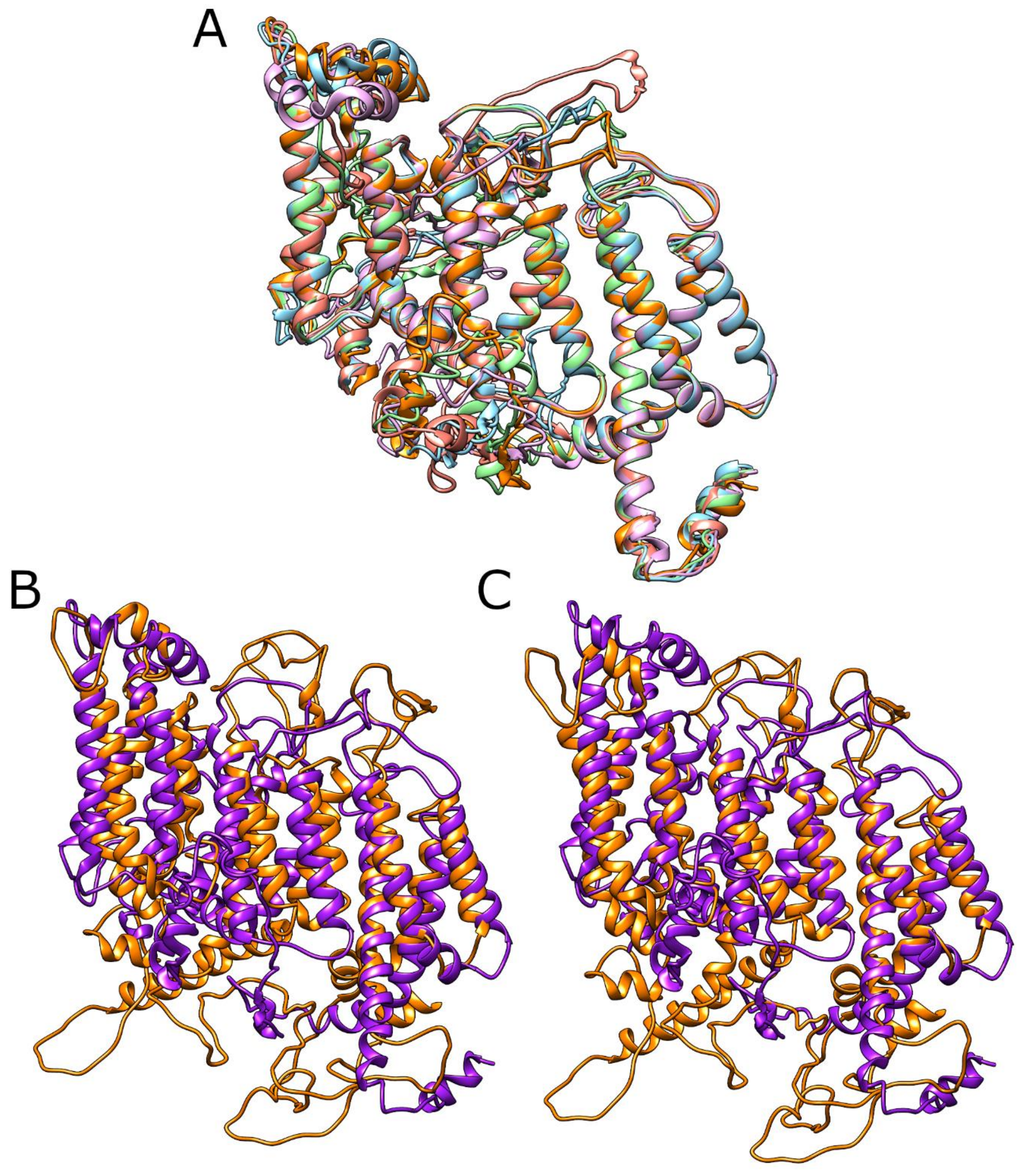

Supplementary Figure 2: Modelling of Piezo1 N-terminal residues by I-TASSER. (A) Top 5 models of Piezo1 N-terminal produced by I-TASSER, superimposed on each other. Model 1 is orange, model 2 is blue, model 3 is pink, model 4 is green, and model 5 is red. **(B)** Top-scoring I-TASSER model (orange) aligned with the corresponding region of our Piezo1$_{full}$ model (purple). All modelled residues (1-576) were used for alignment (RMSD 16.93 Å). **(C)** Top-scoring I-TASSER model (orange) superimposed on our Piezo1$_{full}$ model (purple), with alignment by transmembrane helices only (RMSD of aligned regions 10.18 Å).

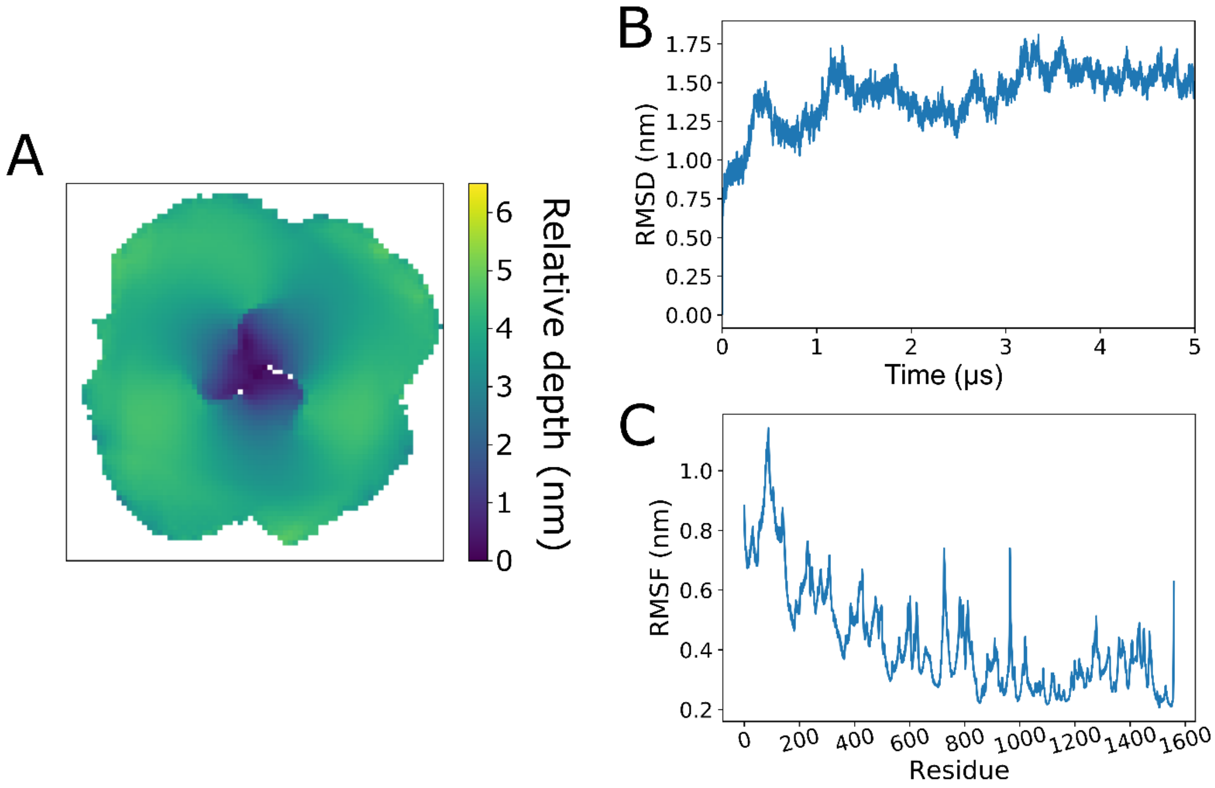

Supplementary Figure 3: (A) Height maps of CG phosphate beads corresponding to the extracellular leaflet in 5 simulations of a Piezo1$_{trunc}$ in a pure PC bilayer, averaged across all repeats. **(B)** RMSD of Piezo1$_{trunc}$ CG beads in a PC bilayer in simulation 1. **(C)** RMSF of Piezo1$_{trunc}$ CG beads in a PC bilayer in simulation 1.

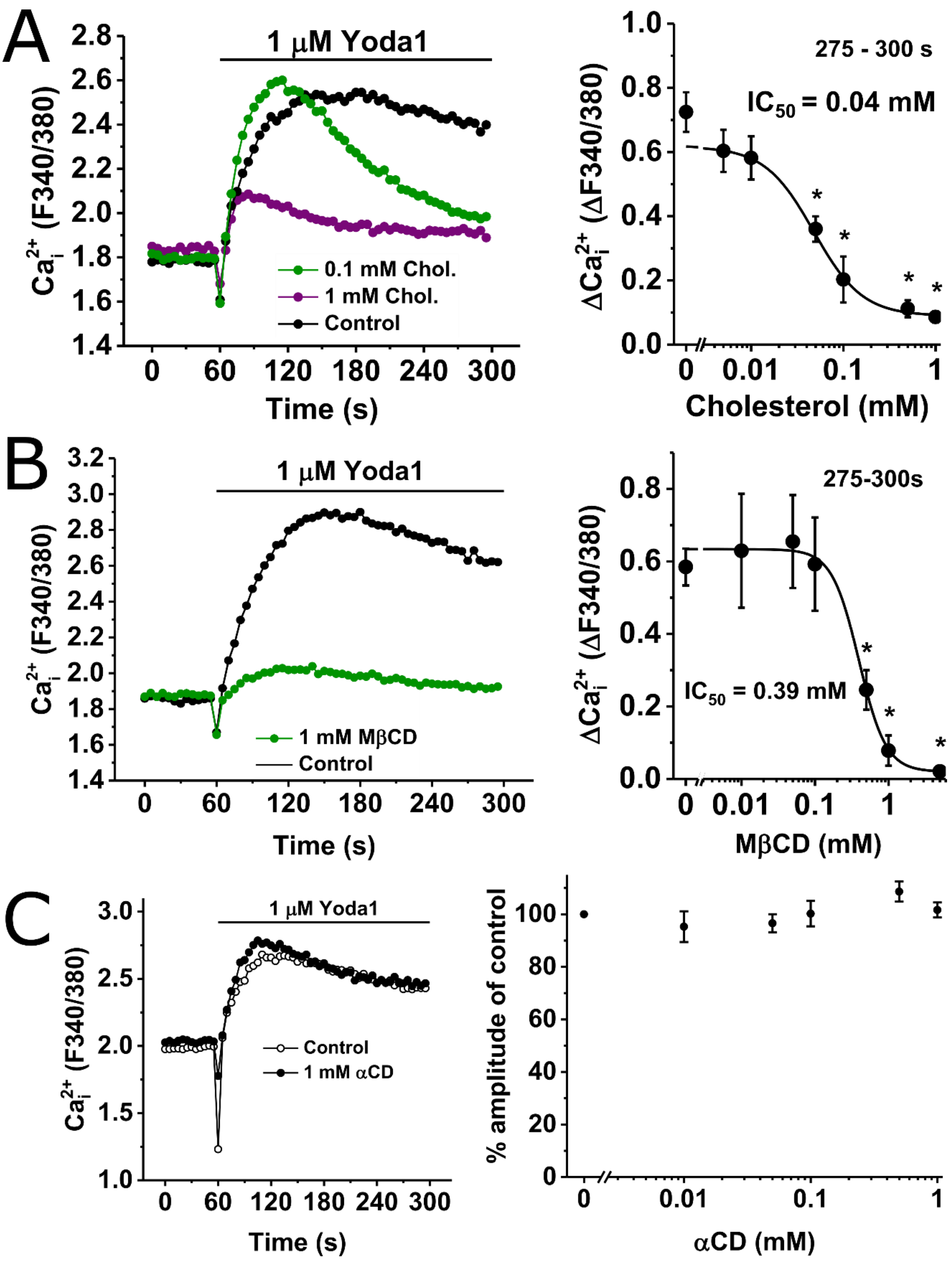

Supplementary Figure 4: Cholesterol concentration modulates Piezo1 activity in HUVECs (A) Left, an example 96-well plate fura-2 measurement of the change in intracellular Ca^{2+} concentration evoked by 1 μM Yoda1 in HUVECs pre-treated with 0 (control), 0.1, and 1 mM cholesterol. Right, mean data for the average amplitude (at 275-300 seconds) of HUVEC responses to Yoda1 with varying doses of cholesterol (0.005-1 mM) displayed as a Hill Equation indicating the 50 % inhibitory effect (IC50) at 0.04 mM. n/N=4/4. (**B**) Left, an example 96-well plate fura-2 measurement of the change in intracellular Ca^{2+} concentration evoked by 1 μM Yoda1 in HUVECs pre-treated with 0 (control) and 1 mM MβCD. Right, mean

data for the average amplitude (at 275-300 seconds) of HUVEC responses to Yoda1 with varying doses of MβCD (5-0.01 mM) displayed as a Hill Equation indicating the 50 % inhibitory effect (IC50) at 0.39 mM. n/N=4/4. **(C)** Left, an example measurement of the change in intracellular Ca^{2+} concentration evoked by 1 μM Yoda1 in HUVECs pre-treated with 0 (control) and 1 mM αCD. Right, mean data of peak amplitudes of HUVEC responses to increasing doses of αCD (1-0.01 mM). n/N=3/4.

Supplementary Table 2: Piezo1$_{trunc}$ residues with cholesterol contacts exceeding cutoff of 60% of maximum contacts.

Y582^{e}	Q703^{e}	D1211^{cef}	M1731^{cef}
V583^{def}	H705^{f}	L1215^{ef}	T1732^{ce}
V589^{def}	Y706^{ef}	M1494^{f}	V1735^{c}
T619^{e}	Y782^{f}	M1495^{f}	E1738^{f}
**Q622**^{bcdef}	L786^{def}	A1499^{f}	M1740^{bcde}
T626^{ef}	S789^{def}	Q1503^{ef}	T1743^{bdf}
L627^{bcde}	F800^{ef}	**F1504**^{bcdef}	K1744^{bcd}
W636^{ef}	H808^{cf}	L1508^{ef}	L1774^{d}
W637^{def}	K811^{cdef}	G1509^{ce}	G1775^{b}
L647^{e}	F986^{e}	T1512^{e}	K1784^{ef}
V650^{e}	**A1019**^{bcdef}	R1671^{cdef}	Y1785^{def}
Y651^{ef}	**T1022**^{bcdef}	T1672^{cef}	F2028^{e}
F655^{ef}	**R1023**^{bcdef}	Y1680^{cef}	W2060^{ef}
F687^{f}	Y1062^{ef}	A1706^{d}	V2141^{f}
A699^{e}	Y1190^{f}	W1717^{f}	W2142^{ef}
L702^{def}	V1208^{ef}	R1728^{cef}	Y2189^{cdef}

The simulated cholesterol concentration in which the residue exceeds cutoff is represented by superscript letters after each residue. Key: b – 5%, c – 10%, d – 20%, e – 30%, f – 40%. Residues which were above cut-off at all simulated cholesterol concentrations are in bold. Residues coinciding with CRAC or CARC motifs are shown in blue. Residues also exceeding cut-off in Piezo1$_{full}$ simulations are underlined.

Supplementary Table 3: Piezo1$_{full}$ residues with cholesterol contacts exceeding cut-off of 60% of maximum contacts.

**L20**[cdef]	F90[f]	G265[ef]	R796[f]	G1509[ef]	Q1944[ef]
**R26**[cdef]	I105[ef]	**T313**[cdef]	Q798[f]	T1512[ef]	F1946[f]
L32[def]	G106[f]	W317[f]	F800[ef]	V1513[ef]	V1948[f]
V33[ef]	T119[f]	Y320[f]	R802[f]	**R1671**[cdef]	Y1955[f]
P42[cef]	**A123**[cdef]	D428[f]	F838[f]	T1672[f]	I1988[e]
W43[cef]	**P124**[cdef]	W533[ef]	F845[f]	Y1680[f]	F2028[e]
**P47**[cdef]	R141[f]	Y588[f]	F936[f]	A1706[def]	W2060[ef]
R58[def]	R187[ef]	Q622[def]	A1019[cef]	L1716[e]	A2077[f]
**R61**[cdef]	R188[ef]	L627[e]	**T1022**[cdef]	W1717[cef]	F2130[e]
A62[e]	T205[ef]	R633[f]	**R1023**[cdef]	M1740[ce]	W2142[f]
C65[de]	S206[ef]	W636[f]	Y1190[e]	T1743[e]	Y2189[ef]
H74[e]	**T209**[cdef]	W637[ef]	V1208[f]	K1744[e]	F2480[f]
A76[def]	L213[f]	R663[f]	D1211[f]	K1784[ef]	
F77[e]	V233[ef]	L778[def]	F1249[f]	Y1785[ef]	
Q78[f]	L235[ef]	**R782**[cdef]	Q1503[f]	F1795[f]	
C80[ef]	A236[e]	L786[e]	F1504[f]	F1932[ef]	
H82[f]	C261[ef]	S789[ef]	L1508[ef]	R1935[f]	

The simulated cholesterol concentration in which the residue exceeds cut-off is represented by superscript letters after each residue. Key: c – 10%, d – 20%, e – 30%, f – 40%. Residues which were above cut-off at all simulated cholesterol concentrations are in bold. Residues coinciding with CRAC or CARC motifs are shown in blue. Residues also exceeding cut-off in Piezo1$_{trunc}$ simulations are underlined. Residues located in the modelled N-terminal region are in an orange box.

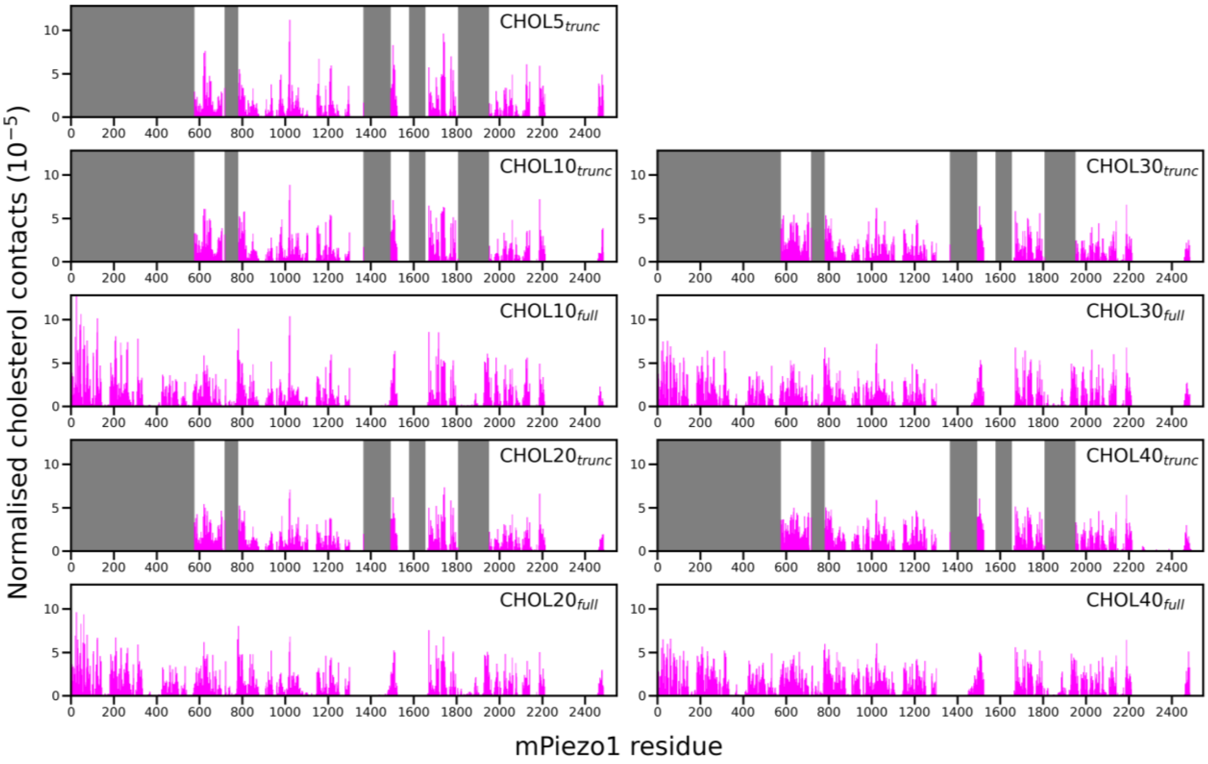

Supplementary Figure 5: Histograms of Piezo1-cholesterol contacts in all simulations. Each histogram is labelled with the corresponding simulation. Grey bars represent the residues missing from Piezo1$_{trunc}$.

Supplementary Table 4: CRAC and CARC motifs in mPiezo1

CRAC	CARC	
^{577}LVTGI**YV**K^{584}	^{113}KDIFNT**T**RLV^{122}	^{1674}RLLRAG**Y**QCV^{1683}
^{618}L**T**LF**Q**VYY**TL**WRK^{630}	^{290}RLFGLKNFV^{298}	^{1724}RPSK**R**FW**MT**AI**V**^{1735}
^{702}**LQ**L**HY**FHR^{709}	^{314}KHA**W**PI**Y**VSPGIL^{326}	^{1762}RYENKPYFPPRI**L**^{1774}
^{840}LPYPR**F**R^{846}	^{537}RQFVKEKLL^{545}	^{1778}KTDSYI**KY**DLV^{1788}
^{862}VCKMLYQLK^{870}	^{602}RLVVYKIV^{609}	^{1797}RSQLLCYGL^{1805}
^{935}V**F**EAVVYRR^{943}	^{629}RKLL**R**VF**WW**LVV^{640}	^{1941}RRL**Q**S**F**C**V**SL^{1950}
^{1058}LCID**Y**PWRWSK^{1068}	^{663}**R**NLTGFTDEQL^{673}	^{1960}RFFHDIL^{1966}
^{1076}LIKWLYLPDFFR^{1087}	^{709}RPFMQLTDL^{717}	^{1971}RAATDVYALMFL^{1982}
^{1252}VCTVKGYYDPK^{1262}	^{796}**RIQ**V**F**V**R**RLL^{805}	^{2035}RALYLRKTVL^{2044}
^{1602}LSTGYNTR^{1609}	^{811}**K**LVALYTVWVAL^{822}	^{2046}KLAFQVVLVV^{2055}
^{1758}VVLRRYENK^{1766}	^{864}KMLYQLKIV^{872}	^{2071}RMFSQN**A**V^{2078}
^{1776}LEKTDSYI**K**^{1784}	^{915}RKGYPNL^{921}	^{2086}KCIYFAL^{2092}
^{1950}LAQSF**Y**QPLQR^{1960}	^{979}KYFINFF**F**YKFGL^{991}	^{2098}RCGYPTRIL^{2106}
^{2032}VIDRALYLRK^{2041}	^{1005}RMNFMVIL^{1012}	^{2112}KKYNHLNL^{2119}
^{2078}VAQLWYFVK^{2086}	^{1031}RLWPNYCLFLTL^{1042}	^{2126}RLVP**F**LVEL^{2134}
^{2092}LSAYQIR^{2098}	^{1147}RSYLDMLKV^{1155}	^{2183}KKKIVK**Y**GMGGL^{2194}
^{2333}VEYTNEK^{2339}	^{1159}RYLFWLVLVV^{1168}	^{2231}KLGGYEPL^{2238}
^{2371}LFPKYIR^{2377}	^{1176}RISIFGLGYLL^{1186}	^{2318}RFTWNFQRDL^{2327}
^{2521}LEEELYAK^{2528}	^{1294}RRIFLSHYFL^{1303}	^{2422}KASDFLEWWV^{2431}
	^{1318}RGFALYNAANL^{1328}	^{2505}KLCQDIFLV^{2513}

Residue numbers are indicated. Residues with significant cholesterol interactions in any simulation are in bold and colored magenta.

Supplementary Table 5: $Piezo1_{trunc}$ residues with PIP_2 contacts exceeding cut-off of 70% of maximum contacts.

L577[ef]	**R844**[abcdef]	Q1496[e]
T579[acdef]	**R846**[abcdef]	**R1497**[abcdef]
**K584**[abcdef]	**R942**[abcdef]	K1727[abcde]
L627[cef]	R949[bcde]	R1728[abce]
**W628**[abcdef]	**R1023**[abcdef]	Q1952[b]
**R629**[abcdef]	**R1024**[abcdef]	R2040[acd]
**K630**[abcdef]	**R1025**[abcdef]	K2183[acde]
**R633**[abcdef]	**R1031**[abcdef]	K2184[abde]
H708[df]	K1201[c]	K2185[acde]
**R709**[abcdef]	**R1204**[abcdef]	G2188[c]
**R782**[abcdef]	**H1493**[abcdef]	R2517[cd]

The simulated cholesterol concentration of systems in which the residue exceeds the cutoff is indicated by superscript letters as follow: a – 0%, b – 5%, c – 10%, d – 20%, e – 30%, f – 40%. Residues which were above the cutoff in all the $Piezo1_{trunc}$ systems are in bold. Residues also exceeding cut-off in $Piezo1_{full}$ simulations are underlined.

Supplementary Table 6: Piezo1$_{full}$ residues with PIP_2 contacts exceeding cut-off of 70% of maximum contacts.

T56[ce]	**R466**[acdef]	**<u>R1024</u>**[acdef]	R1924[acd]
**G57**[acdef]	**<u>R629</u>**[acdef]	**<u>R1025</u>**[acdef]	H1925[ac]
**R58**[acdef]	**<u>K630</u>**[acdef]	<u>K1201</u>[ad]	**K1929**[acdef]
L60[ac]	**L632**[acdef]	**<u>R1204</u>**[acdef]	S1930[cd]
**R61**[acdef]	**<u>R633</u>**[acdef]	R1891[acdf]	K1931[acdf]
**R144**[acdef]	F711[cde]	R1892[acdf]	**R1933**[acdef]
R151[ef]	G724[ce]	R1893[acdf]	**R1935**[acdef]
**K186**[acdef]	**R726**[acdef]	K1894[cdf]	**K1937**[acdef]
**R187**[acdef]	**<u>R844</u>**[acdef]	R1920[acde]	R1941[acd]
R348[def]	<u>R846</u>[c]	**K1921**[acdef]	R1942[acdf]
K349[ef]	**<u>R1023</u>**[acdef]	R1922[cdf]	

The cholesterol concentration of systems in which the residue exceeds the cut-off is indicated by superscript letters as follow: a – 0%, c – 10%, d – 20%, e – 30%, f – 40%. Residues which were above the cut-off in all the Piezo1$_{full}$ systems are in bold. Residues also exceeding cut-off in Piezo1$_{trunc}$ simulations are underlined. Residues located in the modelled N-terminal region are in an orange box.

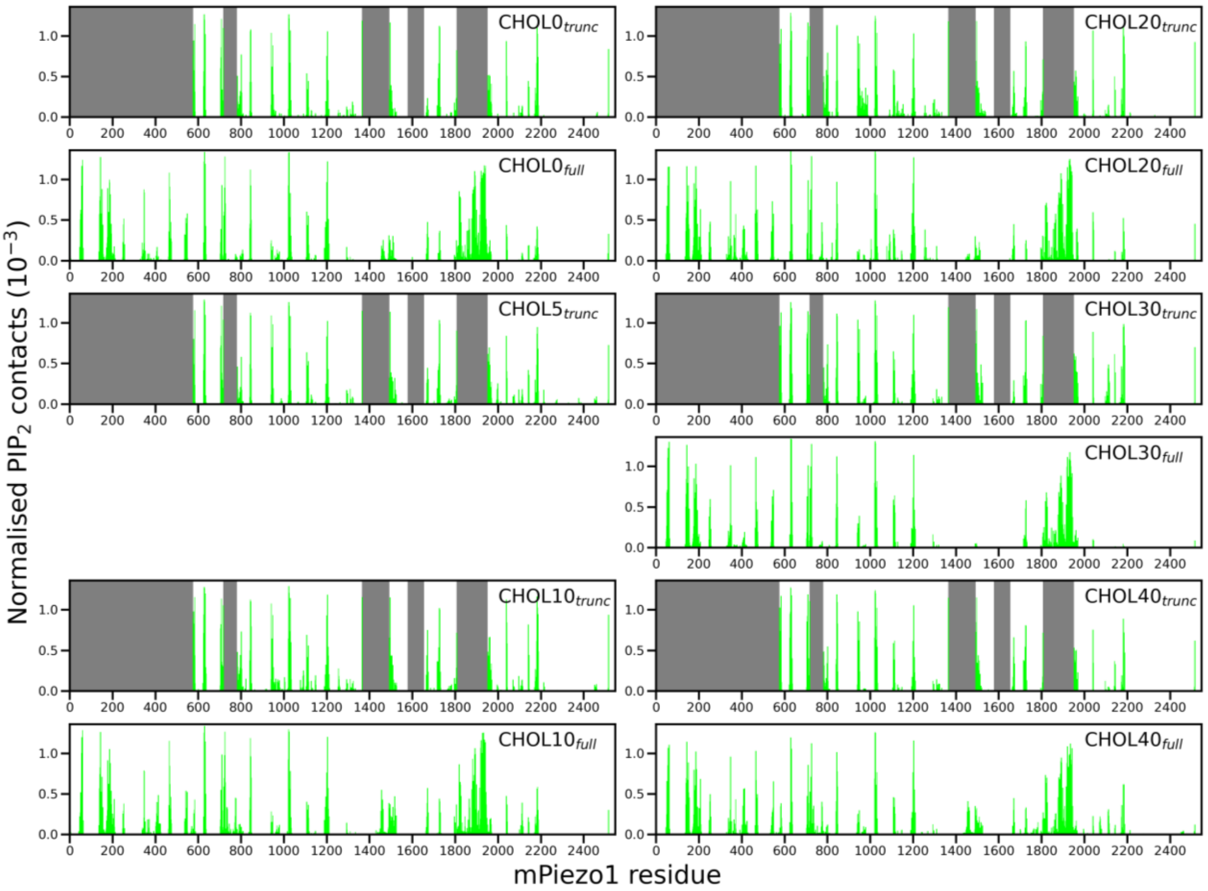

Supplementary Figure 6: Histograms of Piezo1-PIP$_2$ contacts in all simulations. Each histogram is labelled with the corresponding simulation. Grey bars represent the residues missing from Piezo1$_{trunc}$.

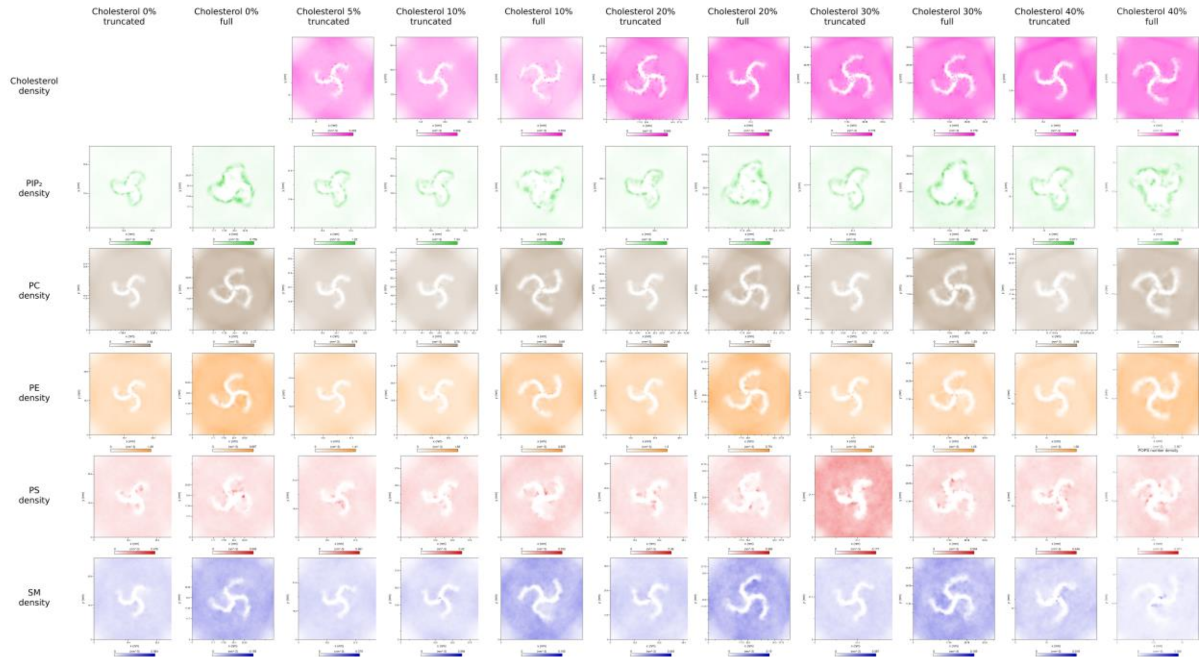

Supplementary Figure 7: Matrix of lipid density maps for all simulations conducted. Lipids are color coded as cholesterol – magenta, PIP$_2$ – green, POPC – tan, POPE – orange, POPS – red, SM – blue.

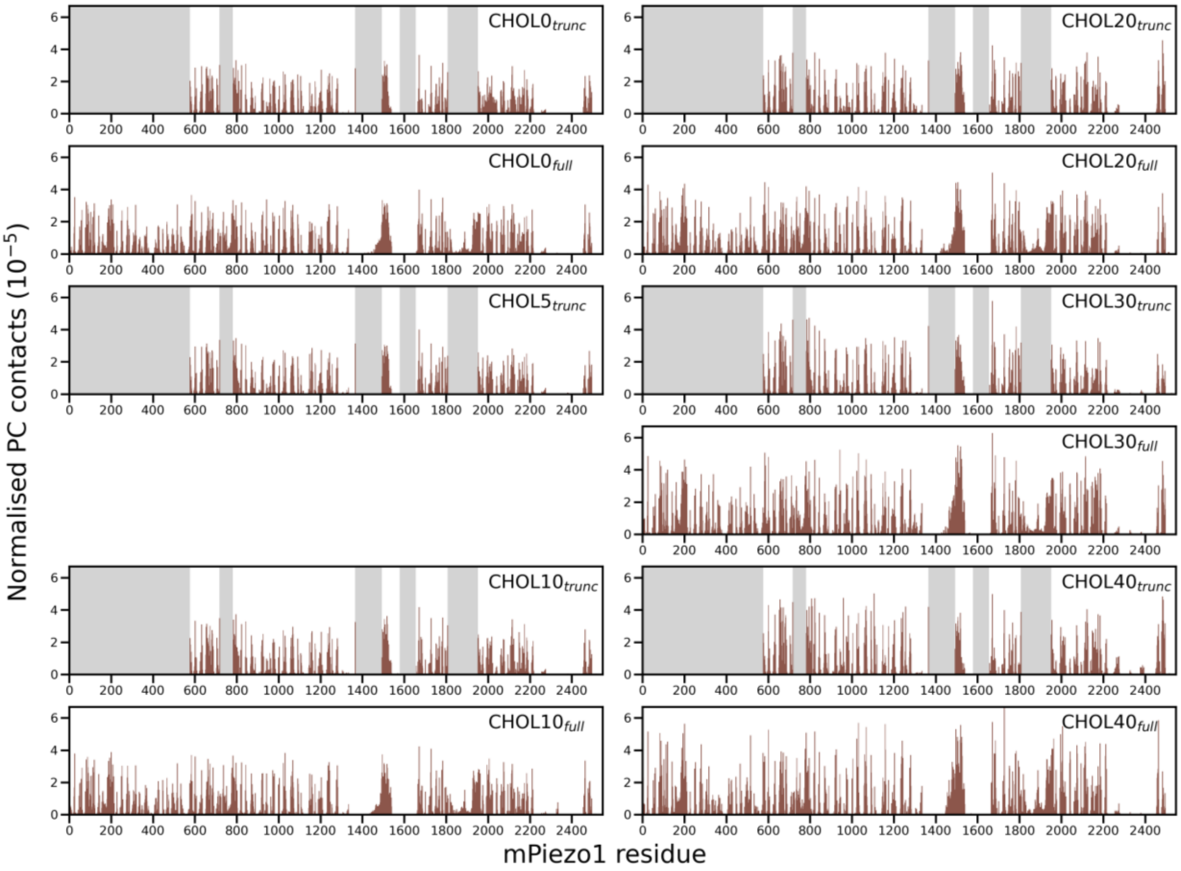

Supplementary Figure 8: Histograms of Piezo1-POPC contacts in all simulations. Each histogram is labelled with the corresponding simulation. Grey bars represent the residues missing from Piezo1$_{trunc}$.

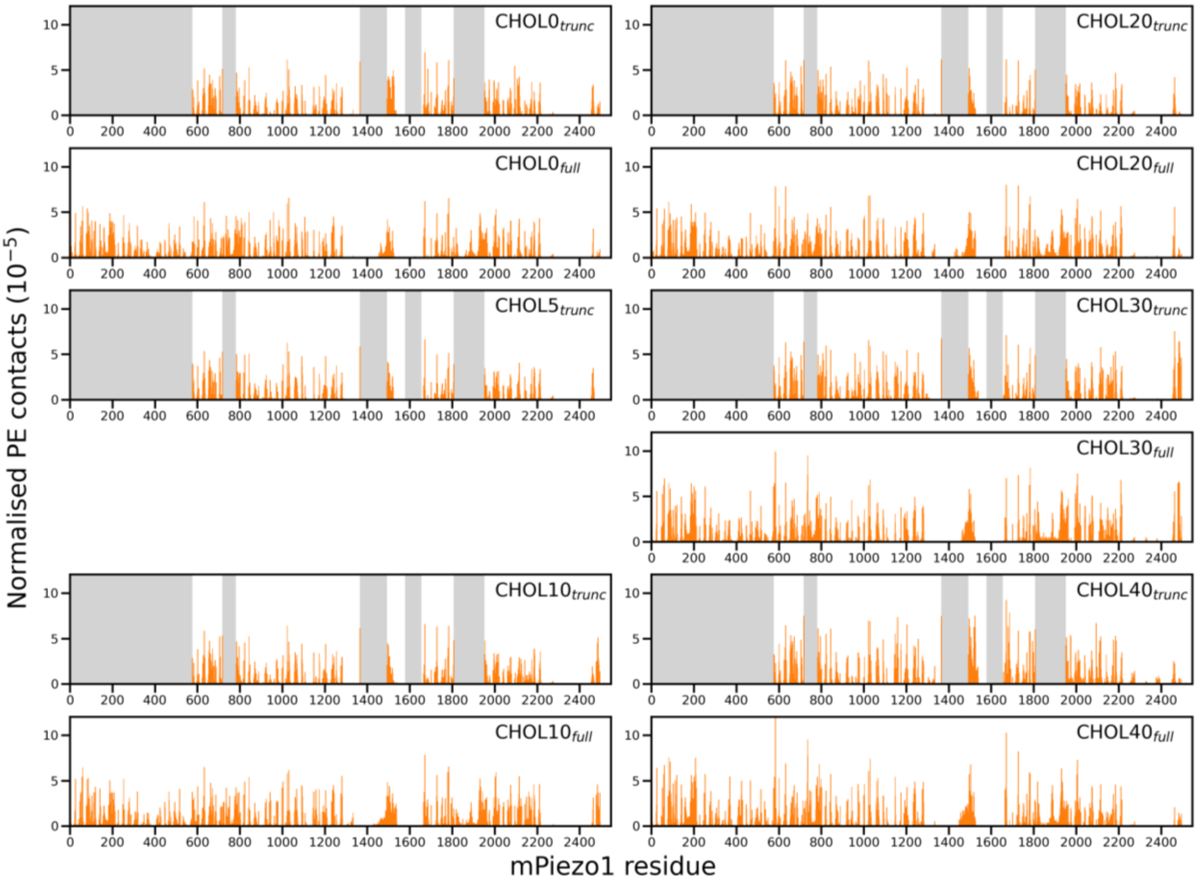

Supplementary Figure 9: Histograms of Piezo1-POPE contacts in all simulations. Each histogram is labelled with the corresponding simulation. Grey bars represent the residues missing from Piezo1$_{trunc}$.

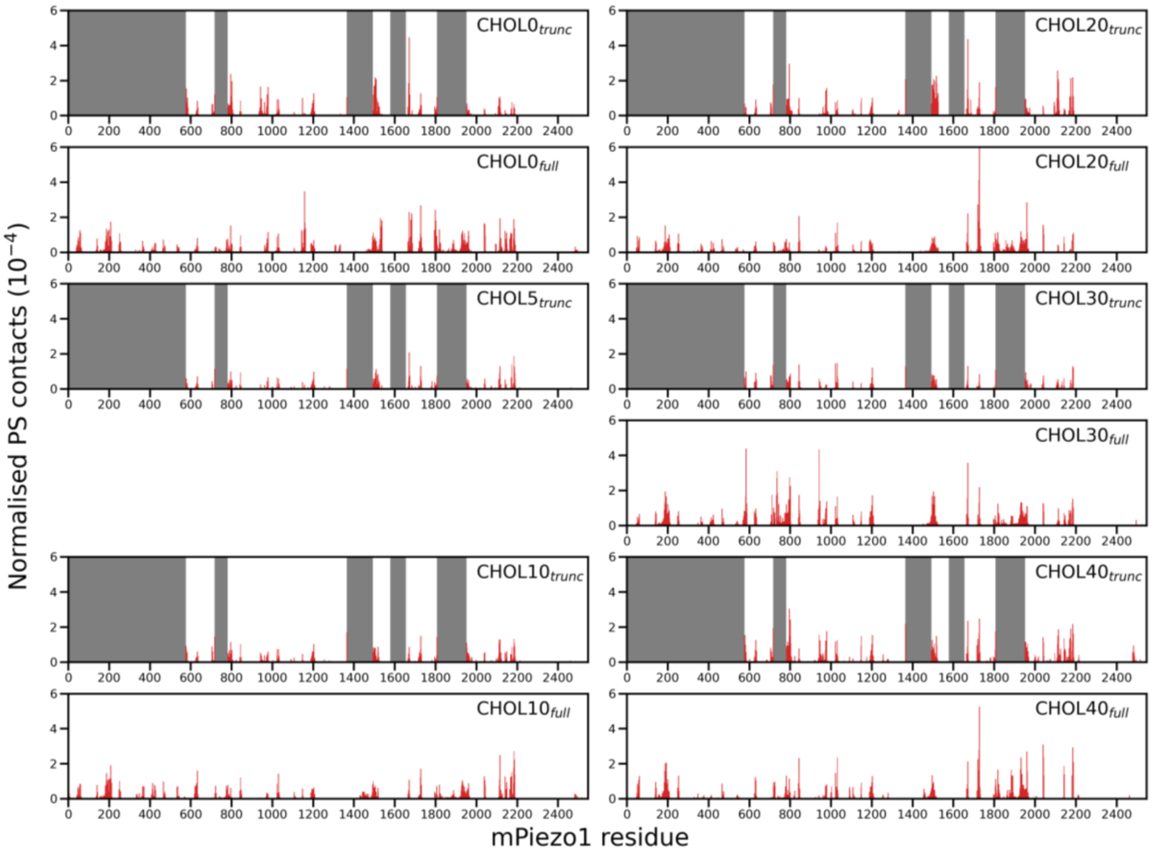

Supplementary Figure 10: Histograms of Piezo1-POPS contacts in all simulations. Each histogram is labelled with the corresponding simulation. Grey bars represent the residues missing from Piezo1$_{trunc}$.

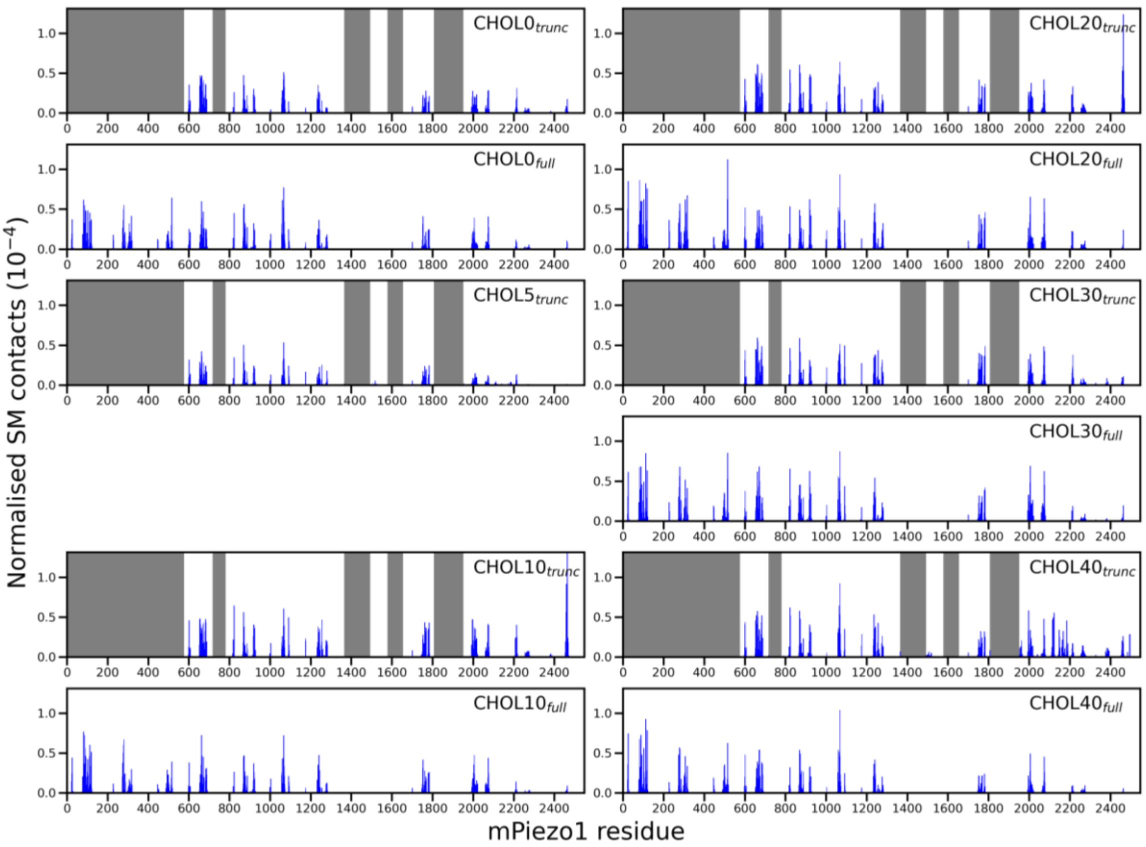

Supplementary Figure 11: Histograms of Piezo1-SM contacts in all simulations. Each histogram is labelled with the corresponding simulation. Grey bars represent the residues missing from Piezo1$_{trunc}$.
